# TrAp: a tree approach for fingerprinting subclonal tumor composition

**DOI:** 10.1093/nar/gkt641

**Published:** 2013-07-27

**Authors:** Francesco Strino, Fabio Parisi, Mariann Micsinai, Yuval Kluger

**Affiliations:** ^1^Department of Pathology, Yale University School of Medicine, New Haven, CT 06520, USA, ^2^NYU Center for Health Informatics and Bioinformatics, New York University Langone Medical Center, 227 East 30th Street, New York, NY 10016, USA and ^3^Yale Cancer Center, New Haven, CT 06520, USA

## Abstract

Revealing the clonal composition of a single tumor is essential for identifying cell subpopulations with metastatic potential in primary tumors or with resistance to therapies in metastatic tumors. Sequencing technologies provide only an overview of the aggregate of numerous cells. Computational approaches to de-mix a collective signal composed of the aberrations of a mixed cell population of a tumor sample into its individual components are not available. We propose an evolutionary framework for deconvolving data from a single genome-wide experiment to infer the composition, abundance and evolutionary paths of the underlying cell subpopulations of a tumor. We have developed an algorithm (TrAp) for solving this mixture problem. *In silico* analyses show that TrAp correctly deconvolves mixed subpopulations when the number of subpopulations and the measurement errors are moderate. We demonstrate the applicability of the method using tumor karyotypes and somatic hypermutation data sets. We applied TrAp to Exome-Seq experiment of a renal cell carcinoma tumor sample and compared the mutational profile of the inferred subpopulations to the mutational profiles of single cells of the same tumor. Finally, we deconvolve sequencing data from eight acute myeloid leukemia patients and three distinct metastases of one melanoma patient to exhibit the evolutionary relationships of their subpopulations.

## INTRODUCTION

The mechanisms of cancer evolution and metastatic onset are still largely unknown. The diversity, complexity and evasive nature of tumor biology are central reasons for the seemingly slow progress in the cure of most cancer types, particularly in controlling the ability of tumor populations to spread. Tumor populations are dynamic aggregates of constantly evolving subclones, each carrying a variety of genomic aberrations (1–35). This genetic heterogeneity is often associated with differences in the biological behavior of different cell subpopulations. Some of these subclones are likely to be the primary instigators of invasion, metastases or relapse following treatment (35–52). An effective characterization of the aggressive potential of tumors at early stages has an enormous potential to guide new clinical interventions and translational research (53–61).

Recently, several efforts have been made to provide a complete genealogical perspective of cancer evolution (62–66). Using fluorescent labeling techniques, or more recently, single-cell sequencing, it is technically possible to separate single cells from tumor samples to investigate their evolutionary patterns (62–71). However, these approaches are limited to either a small number of fluorescent markers (63,72) or to a relatively small number of single cells. On one hand, the identification and selection of uncharacterized subclones in high-throughput experiments is beyond the capabilities of current cell-sorting technologies; on the other hand, isolation and profiling of enough single cells to obtain a statistically representative sample of a tumor composed of millions of cells has, currently, prohibitive costs. For this reason, genomics profiling of tumors still relies on pooling to provide global averaged signals over the subclonal population within a tumor sample (73–76). Computational methods for identifying subclones, quantifying their relative abundance and monitoring their emergence and dynamics could prove extremely useful for the analysis of the heterogeneity of these pooled samples. This problem has been often overlooked due to its mathematical complexity.

We present a mathematical approach to de-mix signals from heterogeneous cell populations into their subclonal components and subsequently unveil the underlying dynamic tumor heterogeneity. Our proposed method is related to the problem of blind source separation (77–86), where both the underlying sources and their relative composition are unknown. In contrast to blind source separation methods, we cannot assume that the underlying sources are statistically independent, we have no prior knowledge of the number of sources and we have at our disposal only one mixture of the unknown sources. This mathematical problem has a vast number of solutions and can be addressed only if additional constraints are imposed. Solutions to this problem can be found by applying Bayesian methods such as hierarchical Dirichlet Processes (66,87). While such approaches typically produce plausible solutions to the problem, they require knowledge of several parameters and prior distributions, which are often not easy to calibrate. Futhermore, stochastic methods are not guaranteed to find the optimal solution(s) to the problem and may miss many solutions. Herein, we introduce biologically meaningful constraints to dramatically reduce the number of solutions to the problem, and we provide an algorithm to find all solutions of this reduced problem. In detail, we assume that tumor cell populations develop in a parsimonious evolutionary process. Furthermore, based on empirical observations, we introduce a sparsity constraint that limits the number of subpopulations. Distinctively from the standard problem of phylogeny (88–99), where each species is observed and measured separately, and differently from cases where multiple aggregate samples have been measured (100–106), our methodology, which we term Tree Approach to Clonality (TrAp), is specifically designed to deconvolve a **single** aggregate signal into its different subclonal components. TrAp incorporates biologically motivated constraints, which allow us to infer (i) the composition of the subclones in a single aggregate sample, (ii) the abundance of each subclone and (iii) the evolutionary path of the subclones.

The article is organized as follows: we first define the subclonal deconvolution problem and we present an efficient algorithm for finding all its solutions in the ‘Results’ section. Using *in silico* simulated data we verify that the algorithm is able to correctly deconvolve mixed subclonal populations and that the method is robust to realistic measurement errors. Further, the solution is often unique when the number of populated subclones is moderate. In addition, we also show that TrAp can correctly deconvolve random mixtures of karyotypes of several cells from the same tumor biopsy or from mixture of sequences generated in a study involving somatic hypermutations (SHMs) in B cells. We subsequently compare the mutation profiles of 20 Exome-Seq single-cell experiments to those inferred using an aggregate signal generated by exome sequencing from the same renal cell carcinoma tumor. We then apply the TrAp algorithm to study the response to chemotherapy of eight acute myeloid leukemia (AML) patients. Finally, we apply TrAp to Exome-Seq data from three metastases from three distinct body compartments and compare their subclonal compositions and evolutionary histories.

### The subclonal deconvolution problem

We consider a population of cells where each cell can be described by a binary vector, which we call **genotype**. Each element of the genotype vector has an aberration state modeled as a binary variable, e.g. the presence/absence of a mutated nucleotide in a specific genomic position or the presence/absence of a specific copy number variation event in a specific locus. For each cell, the *i*-th element of the genotype vector is 1 if the *i*-th aberration is present in the cell and 0 if the aberration is absent. A **subclone** is a collection of all cells that have identical genome-wide aberration profile. A subclone is **populated** in the sample if the fraction of cells sharing the subclone’s genome is >0 and can be detected by the experiment.

We define the **subclonal deconvolution problem** as the task of de-mixing an aggregate measurement into a linear combination of (unknown) subclonal genotypes. The only information that is required as input is the **aggregate frequency vector y**, whose elements *y_i_* correspond to the frequency of the *i*-th aberration in the sample cell population. For efficiency, we remove from the genome all genotype entries *k* that are homogenous within the population (i.e. 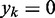 or 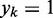), as they do not need to be deconvolved. Next, to ensure that the aberration-free noncancerous cells (wildtype) are included in the solution of the problem, we add one dummy aberration to all the normal and cancerous cells in the sample. By construction, the aggregate frequency of this dummy aberration *y*_1_ is equal to 1. Finally, without loss of generality, we sort the aggregate frequency vector **y** in descending order such that 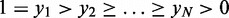, where *N* is equal to the number of aberration events considered including the dummy aberration *y*_1_. The subclonal deconvolution problem can be written in matrix notation as
(1)
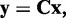

where **C** is a matrix of size 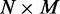 whose elements 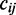 are 1 if aberration *i* is present in subclone *C_j_*_,_ and 0 otherwise; *N* is the size of the vector **y**; *M* is the number of subclones; and **x** is a vector of size *M* where each element *x_j_* corresponds to the frequency of subclone *C_j_* in the sample. We note that, without introducing the wildtype aberration, the wildtype subclone would correspond to a vector of zeros and we would not be able to infer the frequency of the wildtype component using the linear model of Equation (1). Furthermore, because the dummy aberration is present in the wildtype and all other subclones, it follows that (i) 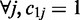 and (ii) 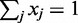. We note that because *M*, **C** and **x** are all unknown in this problem, there is an intractable number of possible solutions. As previously discussed (107), for 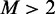, the system is underdetermined and the aggregate signal cannot be uniquely deconvolved by solving the linear system, and it is not even possible to find parsimonious unique solutions using sparse reconstruction methods. However, by introducing additional biologically motivated constraints to the model, we can dramatically reduce the number of possible solutions, such that the problem may have a tractable number of optimal solutions, ideally only one. We therefore seek the family of solutions (**TrAp solutions**) that sequentially satisfy the following constraints:

1. **Evolutionarity**. The subclones are generated in an evolutionary process starting from a subclone with no aberrations. Every subclone is generated from an existing subclone by adding to it a single new aberration event.
2. **Parsimony**. The number of subclones *M* that are generated during the evolution process is minimal.
3. **Sparsity**. The number of populated subclones *P* (i.e. the number of subclones *j* for which 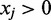) is minimal.
4. **Shallowness**. The depth of the evolutionary tree (i.e. the number of generations) 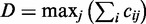 is minimal.

**Figure 1.**
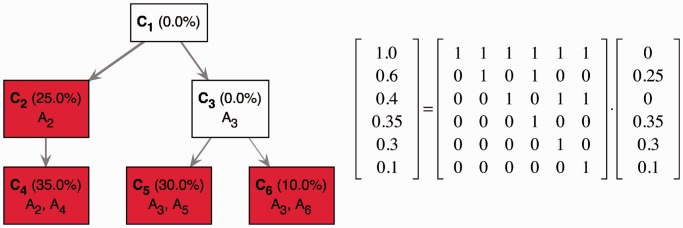
A schema of deconvolution of the mixed signal of four subclones. In this example, the aggregate signal frequency vector **y** on the left side of the matrix-vector equation represents the frequency of five aberrations in the aggregate sample. To allow the heterogeneous mixture of subclones to include normal cells we introduce a dummy aberration that is present in any cell. The frequency of the dummy aberration *y*_1_ is equal to one. The frequencies of the actual five aberrations *A*_2_, *A*_3_, *A*_4_, *A*_5_ and *A*_6_ encoded in the remaining elements of the vector *Y* are given by 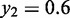, 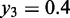, 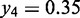, 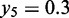 and 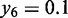, respectively. In this example, the optimal TrAp solution is unique and has four populated subclones: *C*_2_ with aberrations 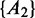, *C*_4_ with aberrations 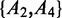, *C*_5_ with aberrations 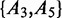 and *C*_6_ with aberrations 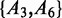. The optimal solution is shown both as an evolutionary tree (left) and in matrix form according to Equation (1) (right), where the tree topology is encoded in the binary matrix and the relative composition of the subclones is represented in the column vector.

A schema of a TrAp solution is shown in Figure 1.

The evolutionarity constraint is used in many biological systems, in particular when studying development of cell populations (90–97,100–102). The parsimony constraint is typically satisfied because the expected probability of a specific aberration event in a nucleotide or a locus is low and it is therefore unlikely that an event occurs more than once and independently in distant subclones. This constraint is the main criterion used to determine the optimality of maximum parsimony methods commonly used in phylogeny (88,89,93,98,105). The parsimony constraint dramatically reduces the number of possible solutions of Equation (1) because it limits the number of subclones *M*. The sparsity constraint is justified by the fact that some subclones may die out or may be too rare to be detected. Also, it has been shown in several studies that few subclones acquire an evolutionary advantage and outgrow the other subclones (5,12,108–112), thus reducing the number of populated subclones. The shallowness constraint is justified as strong genomic instability may not be viable, thus evolutionary trees tend to be shallow and wide rather than deep and narrow.

## MATERIALS AND METHODS

In this section, we describe the procedures used to preprocess input data for the TrAp algorithm.

### Cytogenetic data

The cytogenetic data were obtained from the Mitelman database, which contains 61 846 biopsies as on 15 August 2012. We accessed the database through the Cancer Genome Anatomy Project (CGAP) Web site (113), and we filtered out 29 842 biopsies with uncertain calls (indicated by a ‘?’ in the karyotype data). We focused our search only on aberrations that are binary by nature, namely chromosome deletions and translocations. For each biopsy, we performed 100 *in silico* simulations in which we mixed the subclones using random nonnegative coefficients.

### SHM data

SHM sequencing data were derived from B cells undergoing SHM, a process that leads to high-affinity antibody molecules (114,115). In detail, we analyzed sequences of the V(D)J region extracted from the same germinal center from the sample *11930d16_4print.2*, which was sequenced by Anderson *et al.* (116,117). The sequences were aligned using the alignment tool of the international ImMunoGeneTics information system® (IMGT) (118,119) to properly align the V, D and J regions. We selected eight sequences that were aligned to the same V(D)J sequence 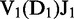. Because these sequences are from the same germinal center and align to the same V(D)J sequence, they are expected to stem from a single naïve B cell and have evolved through the SHM process. From the sequencing experiment, 20 mutated nucleotides were identified. Furthermore, polyallelic mutations were found at position 170, where both 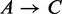 and 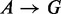 mutations were observed. Next, we considered the seven unique sequences (sequences five and eight were identical) as representatives of the genome of seven different subclones.

### Exome capture sequencing data

Exome-capture data (120) were obtained from a recent clear cell renal cell carcinoma (ccRCC) study (64) and from the melanoma patient YUHUY of the Yale cohort, for which DNA from normal circulating lymphocytes and three tumor metastases (TM1, TM3 and TM4) were subjected to exome-capture Illumina Hi-Seq sequencing (121).

Exome-Seq reads from the aggregate samples of the ccRCC patient were combined and aligned to the human reference genome (assembly hg19) using Bowtie (122) with parameters ‘-n3 -k1 -m20 -l32 –chunkmbs 1024 –best –strata’. The frequency and coverage of each point mutation was computed using VarScan (123). We further selected the mutations that were validated by Xu *et al.* (64) by polymerase chain reaction (PCR) validation (Supplementary Table S3B) and by bioinformatics analysis (Supplementary Table S3A), whose genomic coordinated were realigned to the assembly hg19 using the Lift-Over tool of Galaxy (124).

For the melanoma patient YUHUY (121), we selected 19 mutations that were populated in the normal sample (i.e. 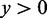), had high sequence coverage (i.e. >200 reads covering the specific nucleotide) and were localized on chromosome 18.

## RESULTS

The results are divided in four parts. In the first part, we describe our novel TrAp algorithm for subclonal deconvolution of aggregate genomic signals consisting of aberrations’ frequencies (e.g. mutational allele frequencies). We show that the TrAp algorithm always identifies at least one solution. Further, we incorporated into TrAp an error model to handle noisy input data as well as an extension for handling situations where each locus can be affected by distinct consecutive aberrations (e.g. nucleotides which can undergo several consecutive mutations such as 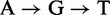, or 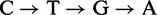). In the second part, we simulate noisy aggregate signals constructed by random linear combinations of simulated subclonal aberration profiles. We used these simulated data to show that TrAp can correctly deconvolve mixtures of evolutionarily related subclones even in presence of levels of noise that are typically found in current genomics experiments. In the third part, we generated realistic aggregate signals by mixing subclonal genomic profiles obtained from cytogenetic analyses using random coefficients. We generate these data separately for each patient and show that, for nearly all aggregate samples, TrAp recovered the subclonal components. Similarly, we generated realistic aggregate signals from somatic hypermutated (SHM) regions from B cells. As we show, SHM is a particularly suitable system for the framework of our TrAp algorithm, which successfully recovered all components from the aggregate signals. In the fourth part, we apply our approach to exome-sequencing experiments. We present an analysis of recent single-cell exome sequencing from a renal cell carcinoma study where, besides a collection of 20 single cells, an aggregate has also been measured. Despite the reported difference between the aggregate and mean aberration profile of the single-cell experiments, TrAp could still identify subclones with co-occurring aberrations consistent with some of the co-occurring aberrations found in direct single-cell measurements. We then apply the TrAp algorithm to study the response to chemotherapy of eight AML patients by comparing the subclonal composition before and after treatment. Finally, we analyze three metastases from separate body compartments of a melanoma patient and compare their inferred evolutionary patterns in a genomic region surrounding the Deleted in Colorectal Cancer (*DCC*) gene.

### A brute-force algorithm for solving the subclonal deconvolution problem

To develop a fast algorithm to solve the subclonal deconvolution problem, we first derive some useful properties that every TrAp solution must satisfy (see Supplementary Note A). First, we note that the evolutionarity and sparsity constraint imply that the evolutionary trees must have exactly *N* clones. We term such a solution an ***N*-solution** and its evolutionary tree an ***N*-tree**. In this setting, mutations happen only once during the evolutionary process and cannot be lost at later stages in evolution. We therefore can define *C_i_* as the subclone for which the *i*-th aberration occurs for the first time.

Next, we note (see Supplementary Note B for derivation and a more detailed description) that, if we consider two subclones *C_i_* and *C_j_* such that 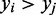 (which implies 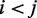 because the **y** vector is sorted), the subclone *C_i_* cannot be a descendant of *C_j_*. This property implies that all evolutionary trees can be generated by a simple iterative procedure, which starts from the wildtype clone *C*_1_ and adds the subclone *C_i_* to all trees generated in the step *i* – 1 (Supplementary Figure S2). The upper bound on the number of possible evolutionary *N*-trees is thus 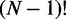, as every subclone *i* can only be the direct descendant of *i* – 1 subclones. This bound is significantly smaller than the number of all possible trees with *N* labeled vertices, which is 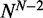 according to Cailey’s formula (125,126).

### The TrAp algorithm

We now rewrite the subclonal deconvolution problem (see Supplementary Note C for derivation) as follows:
(2)
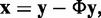

where 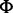 is the parent indicator matrix, whose element 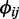 (which we call **parent indicator variable**) is 1 if *C_i_* is the parent of *C_j_* (i.e. if subclone *C_j_* is the result of a single aberration event in subclone *C_i_*), and 0 otherwise. An important corollary of this equation is that the subclone *C_i_* is not populated if and only if
(3)
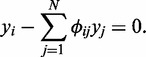

In other words, the clone *C_i_* is not populated when the aggregate frequency *y_i_* of aberration *i* is equal to the sum of the aggregate frequencies of all the direct descendants of the subclone *C_i_*. Therefore, the number of nonpopulated subclones of the *N*-tree encoded by 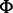 is determined by the number of aberrations *i* that satisfy Equation (3). Moreover, to satisfy the sparsity constraint of a solution, we do not need to know the topology of the whole evolutionary tree, but only the subset of rows of the matrix 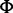 that satisfy Equation (3). We now leverage on this property to efficiently generate sparse solutions to the subclonal deconvolution problem.

**Figure 2.**
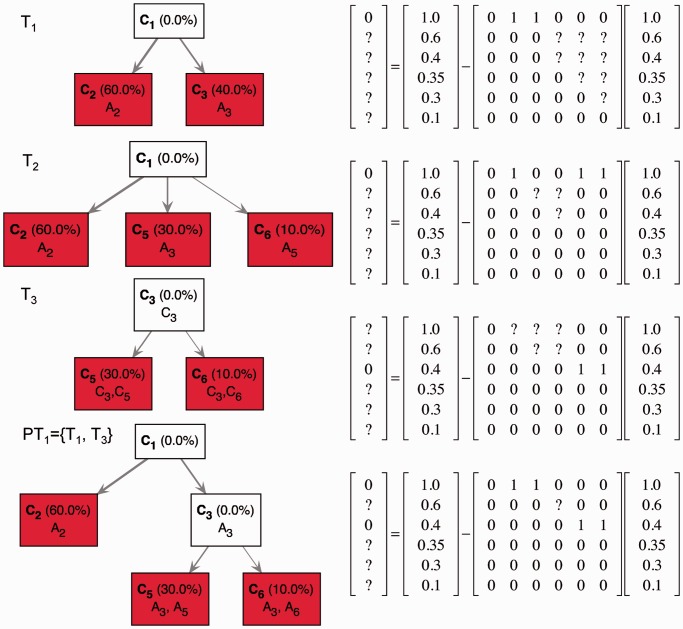
Identification of first-generation trees. In this example, the aggregate signal frequency vector 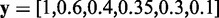 is consistent with three first-generation trees 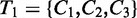, 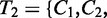 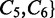 and 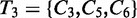. Each first-generation tree is visualized as a matrix equation 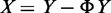 according to Equation (2) (left) and as a partial evolutionary tree (right). In the bottom row, the partial tree *PT*_1_ given by the union of the partial trees *T*_1_ and *T*_3_ is shown. Question marks indicate values that are unknown as they are not specified by the first-generation tree or by the partial tree.

First, we group each subset of subclones that satisfy Equation (3) into a **first-generation tree** *T_i_*, which we define as the subset of subclones 
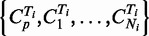
 such that the subclone 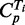 is not populated (i.e. 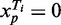) and that *N_i_* subclones 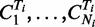 are its direct descendants. Each first-generation tree is represented by a row of the 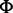 matrix. For example, there are three first-generation trees for the aggregate signal 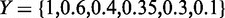, namely 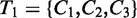, 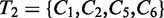 and 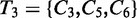 (Figure 2). We note that the optimal TrAp solution for this example contains the first-generation trees *T*_1_ and *T*_3_ (Figure 1). Furthermore, a 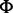 matrix associated with a first-generation tree must follow the evolutionary constraints previously described (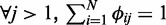), and thus, the first-generation tree also gives complete information about the columns of 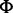 corresponding to the direct descendant subclones 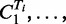 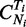. For example, the first-generation tree 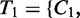 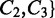 implies that 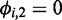 and 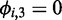 for every 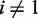 (Figure 2).

Next, we define a **partial tree** as a collection of first-generation trees 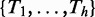 that can jointly be contained in a full evolutionary tree. Because each first-generation tree can be represented by a row of the 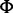 matrix, a partial tree that is comprised of *h* first-generation trees can be represented by *h* rows of the 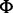 matrix. In the example above, the partial tree that contains the first-generation trees *T*_1_ and *T*_3_ is represented by rows 1 and 3 of the matrix 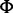 in the bottom panel of Figure 2. Similarly, to first-generation trees, the matrix 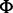 associated with a partial tree must follow the evolutionary constraint, which implies that not all combinations of first-generation trees give rise to partial trees. In the example above, the partial trees *T*_1_ and *T*_3_ can be combined to generate the partial tree 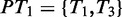 (Figure 2 bottom), whereas the pairs 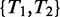 and 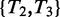 cannot be combined to generate partial trees. Therefore, in the example above, the possible partial trees are 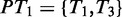, the empty partial tree 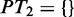 and the partial trees 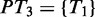, 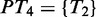 and 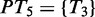. Moreover, we note that all *N*-trees that contain a partial tree comprising of *h* first-generation trees have *N* – *h* populated subclones. This implies that TrAp solutions, which must satisfy the sparsity constraint, must also contain one of the partial trees comprising the maximum number of first-generation trees. In the example above, the optimal TrAp solution (Figure 1) contains the partial tree *PT*_1_, which is the only partial tree comprising two first-generation trees.

**Figure 3.**
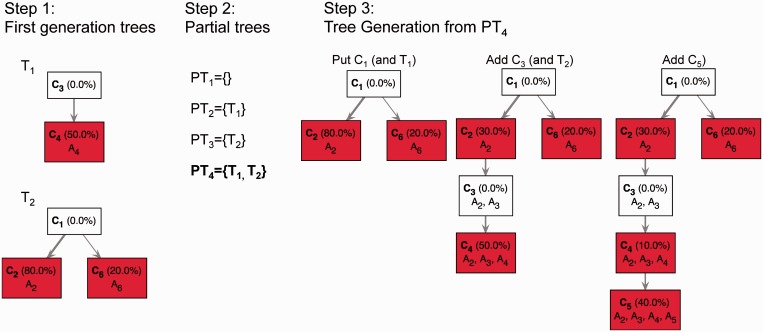
Illustration of the usage of first-generation trees and partial trees for deriving the TrAp solution of a mixture of four subclones. In this example, five aberrations were measured from an aggregate sample and their frequencies were 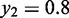, 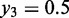, 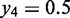, 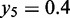 and 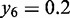, respectively. The dummy measurement 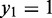 was also added to generate the aggregate signal frequency vector 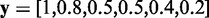. In the first step, TrAp identifies all first-generation trees, namely 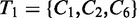 and 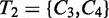. In the second step, TrAp generates the possible partial trees, namely 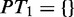, 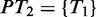, 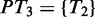 and 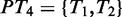, and consequently selects only 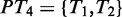, as it is the only partial tree that contains a maximum number of first-generation trees. In the third step, TrAp generates evolutionary trees starting from the partial tree 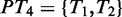. To complete the evolutionary tree starting from *PT*_4_, the subclone *C*_1_ is positioned as the root of the tree. Because *C*_1_ is part of the first-generation tree *T*_1_, the subclones *C*_2_ and *C*_6_ are automatically added as direct descendants of *C*_1_. Next, *C*_3_ is added as a direct descendant of *C*_2_. Because *C*_3_ is part of the first-generation tree *T*_2_, *C*_4_ is automatically added as direct descendant of *C*_3_. Finally, *C*_5_ is added as a direct descendant of *C*_4_, generating the optimal TrAp solution to the subclonal deconvolution problem. We remark that the optimal solution generated by the TrAp algorithm is equal to the left solution of Supplementary Figure S1 and to solution 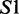 in Supplementary Figure S2.

All TrAp solutions contain the maximum number of first-generation trees, thus the TrAp algorithm dramatically reduces the search space by identifying the optimal partial trees which are then used as starting points for rapidly building all the sparsest solutions to the subclonal deconvolution problem. In detail, the TrAp algorithm solves the subclonal deconvolution problem as follows (Figure 3):

1. Identify all first-generation trees from the aggregate signal vector **y**.
2. Combine all first-generation trees to generate all partial trees.
3. Discard partial trees that do not have the minimum number of populated subclones.
4. Generate all evolutionary trees consistent with the partial trees comprising the maximum number of first-generation trees. This step is done iteratively for each partial tree, similarly to the way described for the brute-force algorithm. The only difference is that, when the parent clone 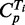 of a first-generation tree 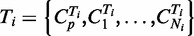 is added to the tree, the subclones 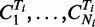 are automatically added as direct descendants of 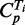.
5. Optimize the shallowness constraint by sorting the generated solutions by the depth of the generated tree.

The performance of the TrAp algorithm is equivalent to the brute-force approach when there are no first-generation trees (i.e. when all subclones are populated), but it becomes superior to a brute-force approach when 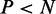. While the brute-force algorithm generates all the evolutionary trees compatible with the input data, the TrAp algorithm generates only the optimal evolutionary tree(s).

### Handling measurement errors

The models presented above show that TrAp is an efficient algorithm for inferring subclonal components from the aggregate measure. In particular, we have shown that in the absence of noise, TrAp returns the exact solution when the underlying subclonal population satisfies reasonable constraints and that the algorithm is always able to find at least one solution. However, experimental measurements are often noisy and can only have finite precision.

In this section, we discuss two approaches to treat noisy input. In both error models, the input to the TrAp algorithm requires an additional vector 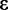 of size *N* whose elements 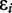 are related to the precision of the measure *y_i_*. The error related to the dummy variable is denoted by 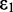 and is set to 0 as 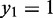 is a constraint of the model and thus 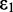 must vanish.

First, we examine the **bound error model** in which we assign a threshold to the error 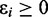 of every underlying aggregate signal 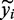 such that each measured signal 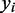 will be in the range 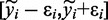. Equation (3) is then modified accordingly and we can state that the subclone *C_i_* defined by aberration *i* is not populated if and only if
(4)
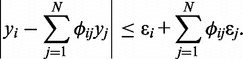

Next, assuming normally distributed measurement errors we implement a normal error model using the confidence intervals to determine whether a subclone is populated. Specifically, we assume that the underlying aggregate signal is normally distributed around the observed signal, i.e. 
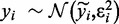
. We substitute each term of the left-hand side of Equation (3) by normally distributed random variables to derive the distribution of the random variable 
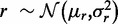
 with mean 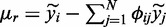 and variance 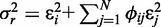. Using the distribution of *r* and a desired confidence level 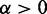 (default 0.05), we can define that clone *C_i_* is not populated if 0 falls within the confidence interval 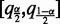, where 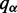 is the α quantile of the distribution of *r*.

Once the error model is chosen, the algorithm generates all optimal TrAp-trees in a similar fashion to the noise-free case. The main difference is that in the first step of the TrAp algorithm, Equation (4) [or a confidence interval on the distribution 
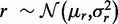
] is used instead of Equation (3) to identify first-generation trees. Moreover, instead of using back-substitution for finding the vector **x**, we solve the nonnegative linear least square problem 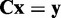 with the constraint 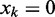 for all nonpopulated subclones *k* associated with the parents of the first-generation trees. This fitting allows us to obtain a value of exactly zero for all nonpopulated subclones and to distribute measurement error more evenly in the vector **x**.

### Extensions and integrations of the TrAp algorithm

The TrAp algorithm was also generalized to deal with nonbinary aberrations (e.g. multiple distinct point mutations at the same nucleotide). This has been done by reducing this generalized nonbinary problem to multiple binary problems that can be solved using the core TrAp algorithm (detailed explanation and derivation are given in Supplementary Note D, Supplementary Figures S3 and S4). This generalized model was used to infer subclonal composition from a mathematical mixture of the SHM data.

Furthermore, the algorithm can be easily modified by imposing additional constraints that need to be satisfied at each step of the iterative tree reconstruction procedure. The contraints can be used to specify the order in which two mutation occur or whether two aberrations must be on separate evolutionary branches (i.e. they will never co-occur). Such constraints are used in the extension of TrAp to nonbinary aberrations (Supplementary Note D). These constraints can also be specified when additional information is available to the user. For example, the aberration state of two nearby nucleotide positions could be observed simultaneously in a read pair. This additional information can be used to determine if two mutations are mutually exclusive or if one is an ancestor of the other (66). Moreover, if multiple samples are available for a given patient, a unique evolutionary tree inferred from one sample can be used to constrain the evolutionary trees of the remaining samples.

The TrAp algorithm by default returns only the solution(s) that optimize all the constraints. In addition, the user can specify parameters to relax the sparsity and shallowness constraints. For example, all *N*-solutions whose number of populated subclones is less than or equal to a desired number can be obtained by retaining more partial trees during the third step of the TrAp algorithm. The solutions produced by TrAp (or the brute-force approach) can then be rescored by more advanced user-defined fitting functions to refine the results. These fitting function may include terms that model the biological system under consideration (77) [e.g. some types of aberrations are more common during SHM (115) or during melanoma development in melanocytes (127)] or terms that model the sampling noise of a given experiment. The TrAp algorithm was used to deconvolve systems of up to 25 aberrations. Although many tumors have larger number of nonsynonymous mutations, the effective number relevant for analyzing the tumor can be significantly reduced. This can be done by (i) considering only a subset of medically relevant genes, e.g. by selecting the first tier defined by Mardis *et al.* (128) or by focusing on expressed genes whose mutations are predicted to be deleterious in proteins or genes that are downregulated relative to normal tissue, (ii) focusing on mutations within selected pathways or (iii) clustering all mutations into groups with similar minor allele frequencies. These reduction approaches allow to identify meaningful aberrations and thus generate trees that are simpler and easier to interpret. Furthermore, studying a smaller number of mutations may be more robust to error and may allow to identify outliers and artifacts in the input data. In the TrAp algorithm, we include a clustering procedure that groups together aberrations with similar frequency (according to the error model chosen) before running the algorithm. More complex clustering methodologies can be applied if replicate samples are available or if multiple samples from the same patient are available. For example, Ding *et al.* (18) applied MCLUST (129,130) and clustering based on Kernel Density Estimation to identify three to five major clusters of minor allele frequencies in three conditions (normal, tumor, relapse) for eight AML patients. Below, we also reanalyzed Ding *et al.* (18) data using the minor allele frequencies of the clusters as input to the TrAp algorithm.

### Implementation of the TrAp algorithm

TrAp was programed in Java. TrAp makes use of the Java Matrix package JAMA (131) for linear regression and code by Josh Vermaas to solve the nonnegative least square problem using JAMA. The Java Universal Network/Graph Framework (132) is used for creating pictorial representations of evolutionary trees. TrAp is released under the GNU Lesser General Public License 3.0 and can be downloaded from the SourceForge repository at the URL http://sourceforge.net/projects/klugerlab/files/TrAp/.

### Deconvolution of simulated noisy aggregates

To confirm that TrAp can correctly infer the subclonal composition from aggregate noisy signals with typical noise levels found in genomic experiments, we performed simulations starting with random *in silico* evolutionary trees with different numbers of aberrations *N* and different numbers of populated subclones *P*. For each tree, we also studied the effect of different magnitudes of measurement errors E and we investigated the operating conditions for which TrAp would correctly identify the true solution.

We performed simulations by sampling genotypes whose size *N* ranged from 1 to 12 and with underlying number of populated subclones *P* ranging from 1 to 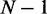. The simulations were repeated for measurement error values E equal to 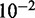, 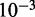 and 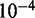. For each combination of these quantities, we performed 1000 runs using *in silico* data as follows: during each run, a random evolutionary tree with *N* aberrations was generated by randomly assigning a parent subclone *C_j_* (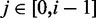) to each subclone *C_i_*. The set of *P* populated subclones was then selected by first including all leaves of the tree and then adding the remaining subclones randomly. The frequency of the populated subclones was randomly assigned and the frequency of the nonpopulated clones was set to zero. Next, the aggregate frequency vector 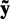 was calculated from the generated tree. Finally, we perturbed each element of 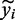 by adding an error 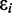 drawn from a uniform distribution 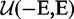. The elements 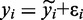 and the error 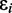 are used as input for the bound error model option of the TrAp algorithm. For each aggregate signal from a random tree, we examined (i) whether the true solution (i.e. the solution associated with the simulated tree), which is by construction one of the possible solutions to the subclonal deconvolution problem, had the minimum number of populated subclones among all solutions (sparsity constraint), (ii) whether the true solution had the minimum number of populated subclones and minimum number of levels of the evolutionary tree among all solutions generated by TrAp (sparsity and shallowness constraints) and (iii) whether the true solution was the only TrAp solution (sparsity and shallowness constraints and uniqueness of the optimal solution).

**Figure 4.**
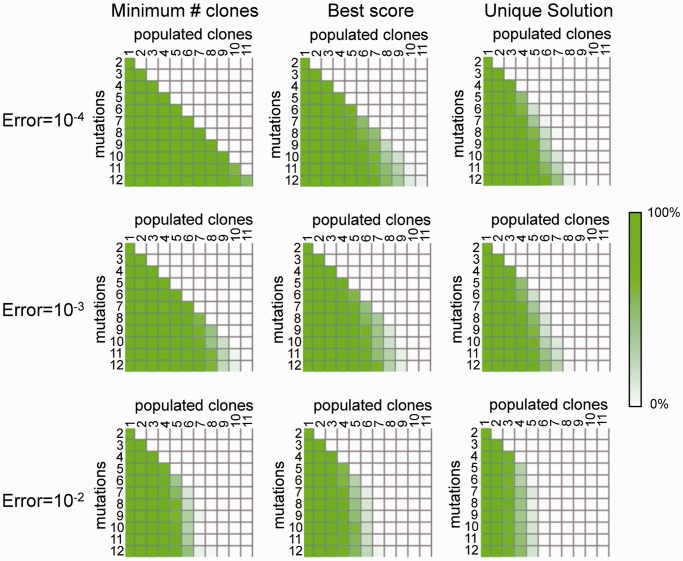
Deconvolution of simulated data. In each table the index of a column represents the number of populated subclones and the index of a row represents the number of mutations. We generated 1000 simulations for any pair of row and column indices (pixel) in these tables. We performed this analysis using different level of noise (error) drawn from a uniform distribution 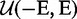. The heatmaps (tables) show the percentage of trees in each cell for which the true solution has the minimum number of subclones (left panel), is a TrAp solution (middle panel) and is the only TrAp solution (right panel) if the best solution is unique.

The results of the simulations show that aggregate signals from sparse trees are deconvolved correctly even in presence of typical noise levels of sequencing experiments (Figure 4). We note that for simulations of nonsparse trees, TrAp generates a large number of possible solutions of which only one is the true solution. Furthermore, in the presence of high levels of noise, the TrAp algorithm identifies a large number of first-generation trees that satisfy Equation (4) and generates solution trees whose number of populated subclones is smaller than the number of populated subclones of the true solution.

### Analysis of simulated mixtures of biological data

#### Deconvolution of mathematical mixtures of karyotyping data from single tumor biopsies

After showing that our approach can correctly deconvolve aggregate signals of subclones with a tree-like genealogy, we sought to investigate whether actual subclonal populations can be charted on evolutionary trees. For this purpose, we analyzed the Mitelman database, consisting of cytogenetic analyses of >60 000 biopsies (see ‘Materials and Methods’). For each tumor type, we counted how frequently the relationships between cancer clones from the same biopsy could be explained by an evolutionary tree that follows the evolutionarity and parsimony constraints (but not necessarily the sparsity and shallowness constraints). We found that almost all biopsies in the Mitelman database can be represented by evolutionary trees (Table 1), with the notable exception of astrocytoma of grades III and IV (Supplementary Figure S5).

**Table 1.** Applicability of the TrAP algorithm for different number of aberration events and underlying subclones

	1	2	3	4	>4
1	100% (19078)	n/a	n/a	n/a	n/a
2	100% (5150)	100% (923)	**0% (3)**	n/a	n/a
3	100% (1830)	100% (367)	94% (83)	**0% (2)**	n/a
4	100% (991)	100% (182)	89% (27)	89% (18)	n/a
5	100% (656)	100% (120)	88% (33)	100% (8)	100% (5)
6	100% (445)	100% (66)	92% (13)	100% (6)	50% (4)
7	100% (333)	100% (58)	89% (9)	25% (4)	100% (2)
8	100% (241)	100% (37)	86% (7)	100% (3)	50% (2)
9	100% (228)	100% (26)	60% (5)	0% (1)	100% (1)
10	100% (174)	100% (14)	100% (2)	n/a	50% (2)
11	100% (196)	100% (25)	67% (3)	67% (3)	n/a
12	100% (156)	100% (16)	100% (3)	0% (1)	50% (2)
13	100% (137)	100% (21)	50% (2)	n/a	100% (1)
14	100% (94)	100% (12)	n/a	100% (1)	100% (1)
>14	100% (152)	100% (22)	57% (7)	25% (4)	25% (4)

Entries where the parsimony constraint cannot be satisfied are shown in bold.

We mathematically mix all karyotypes of each single patient from the Mitelman database and apply the TrAp algorithm for each of these mixtures. The ability of the TrAp algorithm to extract the correct underlying clonal or subclonal components depends on the number of actual components (columns) and the multiplicity of aberrations studied in each mixture (rows). The frequency in which TrAp is able to recover the correct underlying components is shown in percentages. The number of mixtures for a given size of aberration multiplex (row) and given number of actual underlying components (column) is shown in parentheses. Note that when the column index is greater than the row index (entries shown in bold), the parsimony constraint cannot be satisfied.

**Figure 5.**
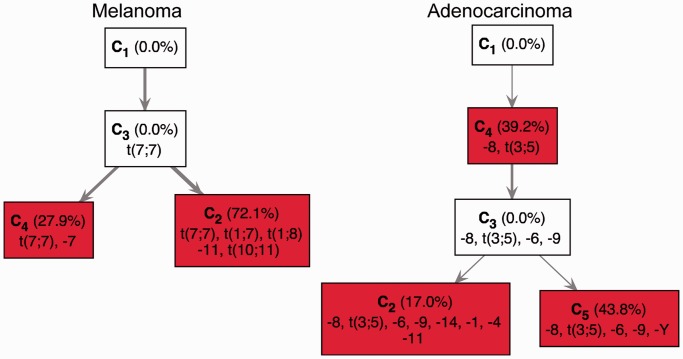
Deconvolution of random mixtures of three subclones. The boxes represent different subclones, each denoted by the list of its aberrations. The aberration profiles of two subclones identified by cytogenetics in a melanoma biopsy (left) and the aberration profiles of three subclones identified in an adenocarcinoma biopsy (right) have been mixed *in silico* using random coefficients. In both cases, the mixtures were successfully deconvolved. Aberrations are grouped within the boxes according to the order of occurrence. The reconstructed evolutionary trees suggest intermediate (white boxes), probably rare, subclones that were not reported in the cytogenetic data.

Next, we investigated whether the TrAp algorithm could uniquely deconvolve mixtures of the cancer subclones within a biopsy. As these aggregate signals are obtained by mixing actual subclonal profiles, we consider these signals to be more realistic than our previous *in silico* simulations. For each biopsy, we generated 1000 *in silico* mixtures by combining the cytogenetic profiles of each subclone using random nonnegative coefficients. We then applied our TrAp method to deconvolve *in silico* mixtures of biopsies. Our results (Table 1) show that 81.5% of the aggregate signals simulated from biopsies with three or more subclones were correctly deconvolved (i.e. in at least one TrAp solution the subclones contained in the biopsies were found and were present in the correct proportions) and that in 67.3% of these simulations there was only one TrAp solution to the deconvolution problem. Moreover, the TrAp algorithm inferred also intermediate nodes in the evolutionary tree that did not correspond to any of the cytogenetic profiles for the biopsy, providing a plausible picture of the evolutionary order in which the aberrations occurred. Figure 5 shows the result of two deconvolution simulations, one from a melanoma sample with two subclonal populations (133) and one from an adenocarcinoma sample with three subclonal populations (134). Interestingly, a small number of biopsies showed more clones than aberrations (shown in bold in Table 1). Albeit a tree-like genealogical relationship can be constructed, these biopsies do not satisfy the parsimony constraint because the number of subclones *M* is greater than the number of mutations *N*. For this reason, their genealogy cannot be reconstructed by the TrAp algorithm or by any other method that makes use of a similar parsimony constraint (88,89,93,98).

#### Deconvolution of a mathematical mixture of SHM data with polyallelic mutations in a single nucleotide

SHM introduces mutations that target the variable regions associated with immune adaptivity in the *Ig* loci. In particular, SHM involves a programmed process of mutations that affects the variable regions of immunoglobulin genes and starts from an initial dividing single cell (a naïve B cell in this case). All descendants of the founder cell accumulate mutations and, at the same time, are subjected to a strong selective pressure. For this reason, SHM is a particularly good biological model system to test our deconvolution method, which imposes tree-like evolutionary constraints.

We considered a data set where 20 mutated nucleotides in the V(D)J region of the *Ig* locus were measured in eight sequences extracted from the same germinal center (see ‘Materials and Methods’ section) (116). This data set was particularly interesting because of the high number of mutations found and because of the presence of polyallelic mutations.

**Figure 6.**
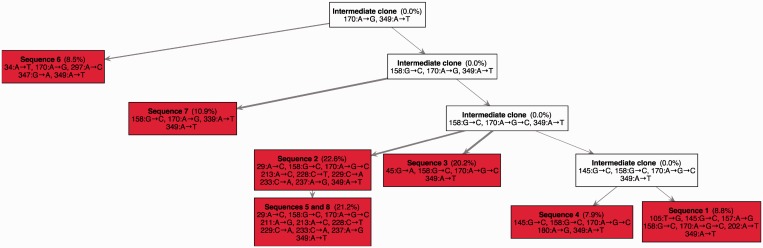
Deconvolution of a random mixture of eight sequences from SHM data. Eight sequences from the *Ig* locus of eight cells extracted from the same germinal center were mixed with the random coefficients given by 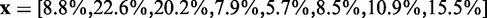. Since sequences five and eight are identical, they are grouped in a single clone whose relative frequency is 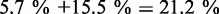. In total, 20 mutated nucleotides were found in the data, and two different mutations were identified at position 170. Mutations are shown using the notation ‘
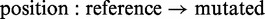
’, e.g., the notation 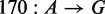 indicates that the nucleotide at position 170 was mutated from Adenine to Guanine. The notation 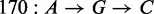 indicates that the nucleotide at position 170 was mutated twice, first from Adenine to Guanine and then from Guanine to Cytosine. In this example, all seven subclones were correctly deconvolved by the TrAp algorithm, the frequency of the subclones was correctly estimated and the solution was unique.

We mathematically mixed the multi-subclonal data and applied our TrAp algorithm taking into account that the SHM scenario consists of nonbinary aberrations. We mixed these subclones using random nonnegative coefficients and performed 1000 simulations. In all simulations, TrAp was able to recover the original sequences and the solution was unique in 65% of the simulations. The TrAp solution of one simulation is shown in Figure 6. However, even if the solution was not always unique, in >97% of the simulations there were only five or less candidate solutions satisfying the evolutionary, parsimony and sparsity constraints, all of which correctly identified at least six out of seven subclones.

In addition to the identification of the underlying subclones, the TrAp algorithm generates evolutionary trees, which represent the B cell lineage during the SHM process. The reconstruction of B cell lineage can provide important insights into the mechanisms that regulate adaptive immunity. B cell lineage reconstruction is generally performed using maximum parsimony constraints (98) using the sequences of several *Ig* loci as input. However, in contrast to these approaches, the TrAp algorithm is able to generate maximum parsimony trees when only the relative frequency of mutations at each nucleotide is available. Therefore, the TrAp algorithm can be used to generate parsimonious evolutionary trees when only partial sequence information is available, e.g. when only short read sequences from a single aggregate sample are available, or when the loci analyzed span a region that is too large to be fully sequenced, or when the loci analyzed are distributed on different chromosomes (e.g. sequences from both Immunoglobulin heavy and light chains).

### Analysis of tumor biopsies

#### Comparison between subclonal aberration profiles inferred from heterogeneous cell populations and single-cell aberration profiles

We analyzed data from a recent study on renal cell carcinoma where two aggregate samples and 20 single cells were isolated from a ccRCC and subjected to exome sequencing. Interestingly, the original study only showed partial similarity between the single cells and the aggregate (64). However, because the single cells and the aggregate used in the experiments are from the same tumor, we sought to investigate whether any subclones inferred by TrAp would share a similar combination of mutations found in any of the single cells.

We applied our TrAp algorithm to the aggregate sample and obtained an evolutionary tree consisting of three main subclones. Due to the lack of extensive validations, we limited ourselves to investigate whether mutations that co-occur in the TrAp solution also co-occur in single-cell samples. We considered the mutations that were validated by bioinformatics analysis [Supplementary Table S3A from Xu *et al.* (64)] and by PCR validation [Supplementary Table S3B from Xu *et al.* (64)]. The fraction of correctly estimated co-occurrences was 0.76 for mutations validated by bioinformatics analysis and 0.74 for mutations validated by PCR.

### AML data

Next, we used our TrAp algorithm to investigate the clonal evolution of eight AML patients. For each patient, three samples (normal, tumor, relapse) were collected and sequenced by Ding *et al.* (18). Minor allele frequencies of somatic mutations were estimated from the sequencing data [Supplementary Tables S5a and S6a–g from Ding *et al.* (18)] and clustered using MCLUST for patient UPN933124 [Supplementary Table S5c from Ding *et al.* (18)] or Kernel Density Estimation for the other seven patients [Supplementary Table S10 from Ding *et al.* (18)]. Since the frequency of each cluster was estimated by the median, we used median absolute deviation and a default scale factor of 1.4826 to estimate confidence intervals under the assumption of an underlying normal distribution (135). The aggregate signal *y_i_* and measurement error 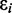 for each cluster of mutations *i* were then estimated as
(5)
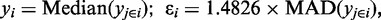

where *y_j_* is the estimated aggregate signal of mutation *j*.

The clonal evolutions inferred by the TrAp algorithm (Supplementary Figures S6–S13) are in agreement with those inferred by Ding *et al.*, who used deductive reasoning to manually derive the subclonal evolution (18). This agreement was expected as all the observations used by Ding *et al.* to generate the evolutionary trees are corollaries of our evolutionarity and sparsity constraints and are therefore automatically enforced by the TrAp method. In addition, the TrAp program listed all evolutionary trees compatible with the input data and provide additional insights on the possible origin of sublclones in the relapses of patient UPN758168 (Supplementary Figure S7) and UPN452198 (Supplementary Figure S10).

### Melanoma data

Finally, we applied our algorithm to investigate evolutionary mechanisms in tumor metastases using exome sequencing data from three tumor metastases (TM1: left lateral chest wall, TM4: pleural cavity and TM3: right axilla) and a matched normal sample (N: left lateral chest wall) of one melanoma patient (121). TrAp can efficiently handle aggregate signal vectors of ∼20 unique frequencies and therefore we perform deconvolution analysis only on one chromosome. We selected chromosome 18, as it contains the tumor suppressor *DCC* gene, which is known to exhibit a high load of mutations only in melanoma (136), in contrast to low expression, loss of heterozygosity or epigenetic silencing in other tumors.

To apply the TrAp algorithm, we first preprocessed the data and selected 19 mutations on chromosome 18 (see ‘Materials and Methods’ section). We labeled each mutation according to the gene affected and the amino acid change caused by the mutation (e.g. the label DCC.L1099H indicates a mutation in the *DCC* gene that causes a mutation from a Leucine to a Histidine at position 1099 in the DCC protein). There are six mutations with 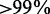 frequency in all samples (including the normal): ADNP2.G54G, ALPK2.I2157V, CD226.S307G, DCC.F23L, NETO1.S481N and SLC39A6.E119D. The only other mutation found in the normal sample was TCEB3CL.S301C, which occurs with frequency 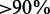 in all samples. Moreover, the mutations ALPK2.R136S, CHST9.S122N, FAM38B.V2463, LAMA1.S1577A, LAMA1.K2002E, MYOM1.T215M, SERPINB10.R246C and SLC14A2.A880T were found in all three tumor samples and shared a similar frequency profile. The mutations DSC3.A28D, DSG1.M11V and IMPACT.D125E were found only in the metastases samples TM3 and TM4 and shared a similar frequency profile. Finally, the mutation DCC.L1099H was found only in the sample TM3.

Since none of the genomic positions analyzed contained polyallelic mutations, we assigned a binary state (normal/mutated) to each selected genomic position and we estimated the aggregate signal and measurement error for each mutation event using a normal approximation as
(6)
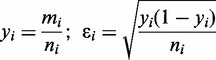

where *n_i_* is total the number of reads covering position *i*, and *m_i_* is the number of reads with a mutation in position *i*. Finally, the **y** and 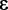 vectors were used as input for the TrAp algorithm. We run the TrAp algorithm using the normal error model option.

**Figure 7.**
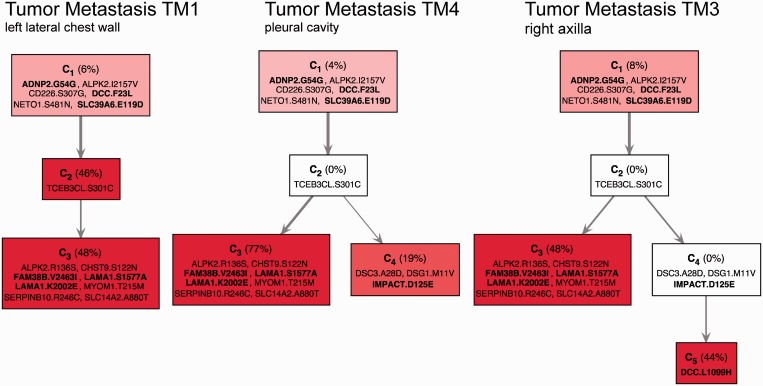
Evolutionary trees inferred from three metastases of a melanoma patient. Each subclone in these trees is represented by a box with a list of mutations that includes only its new mutations (ancestral mutations can be read off by tracing back the mutation lists of all of its ancestors). Mutations are labeled according to the gene affected and the amino acid change caused by the mutation (e.g. the label DCC.L1099H indicates a mutation in the *DCC* gene that causes a mutation from a Leucine to a Histidine at position 1099 in the DCC protein). Highly expressed genes from this patient are indicated in bold. Mutations in the left branch of TM4 are more abundant than in TM1 and TM3. 44% (19%) of the subclones of TM3 (TM4) have mutations in DSC3, DSG1 and IMPACT. The TM3 subclone has an additional mutation in DCC.

Independent runs on the three metastatic samples gave 33 optimal solutions for TM1, 222 for TM3 and only 1 TrAp solution for TM4. These high numbers of solutions are due to the substantial noise of the experiment (in the range 0.005–0.025) and the fact that in samples TM1 and TM3, two of the subclones have similar frequencies and are thus difficult to separate from one another. However, TrAp identified a unique solution in the sample TM4, where the three populated subclones are distributed with significantly different frequencies (Figure 7 middle). Next, we reasoned that the metastatic TM1, TM3 and TM4 samples may share common ancestors and that their evolutionary profiles may be related. We then applied our TrAp algorithm while also imposing that all evolutionary trees must be a subset of one global evolutionary tree. This approach returned a unique solution for each sample, all of which were among the solution sets identified in the previous analyses. We observe that this approach can be very powerful because, in principle, it allows the reconstruction of large trees by combining several snapshots of the related subclonal populations.

The results of the deconvolution are shown in Figure 7. We observe the presence of two main branches. Mutations in the left branch of TM4 (77%) are more abundant than in TM1 and TM3 (48%). We note that Laminin, alpha 1 (LAMA1), a protein that is involved in cell adhesion, is present in the right branch. 44% (19%) of the subclones of TM3 (TM4) have mutations in Desmocollin-3 (DSC3), Desmoglein-1 (DSG1) and Impact RWD Domain Protein (IMPACT). The TM3 subclone also acquires a second mutation in the *DCC* gene (DCC.L1099H-L) in addition to the mutation DCC.F23L, which was hereditary. The novel mutation in the *DCC* gene occurs close to the boundary between the extracellular domain and the transmembrane domain of the protein product. The resulting Histidine amino acid is positively charged, opposed to the Leucine amino acid of the wildtype, which is neutrally charged. Since this change is next to the cell membrane, it may have repercussions on the functionality of the DCC protein product, perhaps causing inactivation, similar to the inactivation caused by loss of heterozygosity and transcript suppression observed in other cancer types.

## DISCUSSION

In the present study, we described the TrAp algorithm, a tool for inferring subclonal composition and abundance from a single aggregate measurement experiment. As we have shown, TrAp is robust to noise and it can deconvolve mixtures where multiple mutations occur at the same locus. TrAp efficiently enumerates all possible solutions that are compatible with the model constraints, thus always identifying the sparsest and most parsimonious solution(s). However, TrAp will also generate trees [cf. Supplementary Equation (S1)] in cases where no tree structure can be inferred. As we have shown, such structures, while deviating from the true underlying population structure, can still capture relevant co-occurrences of mutations that are specific to certain subclones. Further, in contrast to parsimonious neighbor-joining approaches, which rely on sampling single subclones from the population (e.g. single-cell experiments), TrAp uses aggregate experiments as input, thus overcoming the issue of small sampling size, which may be insufficient to cover the whole spectrum of subclones in a sample. We successfully deconvolved systems of up to 25 aberrations. Although this number is not large enough to consider all somatic mutations found in a tumor sample, this problem can be circumvented by clustering aberrations with similar frequencies before running the TrAp algorithm.

The level of characterization achieved by subclonal deconvolution holds high potential for personalized therapies. Possible applications include the classification of subclones in primary tumors, the identification of the seeds of metastases, tracing of resistant subclones especially after drug treatments and developing treatment strategies to eliminate resistant subclones. Furthermore, our proposed model can be applied to other medical problems, such as tracing bacterial or viral paths of adaptation within the infected host, detailed genome-wide reconstruction of the epigenetic differentiation program or class specification in the hematopoietic system or in other systems.

## SUPPLEMENTARY DATA

Supplementary Data are available at NAR Online.

Supplementary Data
