# Supplementary material for: TrAp: a tree approach for fingerprinting subclonal tumor composition
Source: Nucleic Acids Res. 2013 Jul 27;41(17):e165. doi: 10.1093/nar/gkt641 (PMC3783191; doi:10.1093/nar/gkt641)
Supplement: Supplementary Data [file supp_41_17_e165__index.html]

TrAp: a tree approach for fingerprinting subclonal tumor composition — TrAp: a tree approach for fingerprinting subclonal tumor composition — Supplementary Data 

# TrAp: a tree approach for fingerprinting subclonal tumor composition

## 

files

**Files in this Data Supplement:**

- Supplementary Data - pdf file
