# Supplementary material for: TrAp: a tree approach for fingerprinting subclonal tumor composition
Source: Nucleic Acids Res. 2013 Jul 27;41(17):e165. doi: 10.1093/nar/gkt641 (PMC3783191; doi:10.1093/nar/gkt641)
Supplement: Supplementary Data [file supp_gkt641_nar-00196-n-2013-File002.pdf]

Supplementary Data. TrAp: a Tree Approach for Fingerprinting Subclonal Tumor Composition

Francesco Strino [1,†], Fabio Parisi [1,†], Mariann Micsinai [1] and Yuval Kluger [1,*]
[1]Yale University School of Medicine, Department of Pathology
New Haven, CT 06520, USA

Contents

A N-solutions and N-trees **2**

B A brute-force algorithm for solving the subclonal deconvolution problem **3**

C Expressing the subclonal deconvolution problem as a function of the direct descendants **5**

D Generalization of TrAp to non-binary aberrations **6**

Supplementary Figures **10**

References **16**

*To whom correspondence should be addressed. Tel: +1 203 737 6262; Fax: +1 203 785 6486; Email: yuval.kluger@yale.edu.

†The authors wish it to be known that, in their opinion, the first two authors should be regarded as Joint First Authors.

A N-solutions and N-trees

In this section we show that in the subclonal deconvolution problem the evolutionarity and parsimony constraint can always be satisfied by a naïve model in which each aberration event occurs exactly once during evolution (i.e. $M = N$). We call any solution with $M = N$ an ***N*-solution** and its evolutionary tree an ***N*-tree**.

The N-solution optimally satisfies the evolutionary and parsimony constraints. Since all detected aberrations need to occur at least once in the evolutionary tree, the number of subclones must be greater than or equal to the the number of aberration events considered, i.e. $M \geq N$. We note that it is always possible to construct a valid solution for Equation (**1**) of exactly $M = N$ subclones for every aggregate frequency vector $\mathbf{y}$. Specifically, for a cascade-like evolutionary process with no branching, where the wildtype subclone C_1 is the root of the tree and every other subclone C_i is a direct descendant of the subclone C_{i-1}, the solution of Equation (**1**) is given by:

$$\begin{bmatrix} 1 \\ y_2 \\ y_3 \\ \vdots \\ y_N \end{bmatrix} = \begin{bmatrix} 1 & 1 & 1 & \cdots & 1 \\ 0 & 1 & 1 & \cdots & 1 \\ 0 & 0 & 1 & \cdots & 1 \\ \vdots & \vdots & \vdots & \ddots & \vdots \\ 0 & 0 & 0 & \cdots & 1 \end{bmatrix} \begin{bmatrix} 1 - y_2 \\ y_2 - y_3 \\ y_3 - y_4 \\ \vdots \\ y_N \end{bmatrix} \tag{S1}$$

While this cascade-like solution satisfies both evolutionarity and parsimony constraints, this solution is not necessarily optimal with respect to the sparsity constraint and is the least optimal in terms of the shallowness constraint. Furthermore, the existence of this solution guarantees that there is always at least one solution to the subclonal deconvolution problem.

Since we required that the four constraints to the subclonal deconvolution problem must be satisfied sequentially, any solution with $M > N$ subclones will always be less optimal than the solution given in Equation (**S1**), regardless of its sparseness and shallowness. We can thus limit the search space of the TrAp algorithm to N-solutions. For this subset of solutions, the vector $\mathbf{x}$ is of size N and $\mathbf{C}$ is a square matrix of size $N \times N$. Importantly, the index of each subclone C_i indicates the subclone for which the i-th aberration occurs for the first time. Hence, for any TrAp solution there is a one-to-one correspondence between the i-th aberration and the subclone C_i whose index indicates that it evolved from its parent subclone by acquiring the i-th aberration. Moreover, each aberration event of an N-tree occurs exactly once and cannot be reverted during evolution. Each aberration i is thus present only in subclone C_i and its subclonal descendants:

$$y_i = \sum_{j=1}^{N} c_{ij} x_j = x_i + \sum_{j=1}^{N} \alpha_{ij} x_j, \tag{S2}$$

where α_{ij} is the **ancestor indicator variable** that is equal to 1 if subclone C_i is an ancestor of subclone C_j and 0 otherwise. We note that $\forall j > 1, \alpha_{1j} = 1$, which means that the wildtype clone C_1 is always the root of the evolutionary tree, as required by the evolutionarity constraint.

B A brute-force algorithm for solving the subclonal deconvolution problem

We now observe that the relationship between two subclones C_i and C_j such that $i < j$ (which also implies $y_i \geq y_j$ as the vector $\mathbf{y}$ is sorted), must be one of the following: i) C_i is an ancestor of C_j, i.e. $\alpha_{ij} = 1$, $\alpha_{ji} = 0$ and $y_i \geq y_j$; or ii) C_i and C_j are on separate branches, i.e. $\alpha_{ij} = 0$, $\alpha_{ji} = 0$ and $y_i + y_j \leq 1$. This property implies that all evolutionary trees can be generated iteratively by starting with the wildtype clone C_1 and adding the clone C_i at step i to all trees generated at step $i - 1$. In detail, for any tree that can be generated using subclones $C_1, \ldots, C_{i-1}$ we generate a new tree by adding the subclone C_i as direct descendant of subclone C_j for all $j < i$ for which the resulting x_j (calculated using Equation (**2**)) remains nonnegative after adding C_i.

For completeness, if $y_i = y_j$ the subclones C_i and C_j can be either on separate branches or on the same branch. When they are on the same branch, there is an ambiguity regarding the order in which the two aberrations occur (Figure S1). However, in the case that these two subclones are on the same branch, the aberration profile of the ancestor subclone (shown in green in Figure S1) is not informative because this subclone is not populated ($x_a = 0$) and aberrations i and j are both present in the descendant subclone regardless of the order in which they occur. Since these two aberrations cannot be observed separately (i.e. the coefficient x_a associated with the ancestor aberration is zero, whereas the x_d associated with

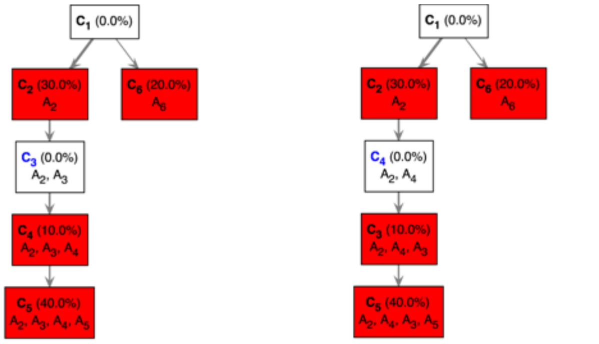

$$\begin{bmatrix} 1.0 \\ 0.8 \\ 0.5 \\ 0.5 \\ 0.4 \\ 0.2 \end{bmatrix} = \begin{bmatrix} 1 & 1 & 1 & 1 & 1 & 1 \\ 0 & 1 & 1 & 1 & 1 & 0 \\ 0 & 0 & 1 & 1 & 1 & 0 \\ 0 & 0 & 0 & 1 & 1 & 0 \\ 0 & 0 & 0 & 0 & 1 & 0 \\ 0 & 0 & 0 & 0 & 0 & 1 \end{bmatrix} \begin{bmatrix} 0 \\ 0.3 \\ 0 \\ 0.1 \\ 0.4 \\ 0.2 \end{bmatrix} \qquad \begin{bmatrix} 1.0 \\ 0.8 \\ 0.5 \\ 0.5 \\ 0.4 \\ 0.2 \end{bmatrix} = \begin{bmatrix} 1 & 1 & 1 & 1 & 1 & 1 \\ 0 & 1 & 1 & 1 & 1 & 0 \\ 0 & 0 & 1 & 0 & 1 & 0 \\ 0 & 0 & 1 & 1 & 1 & 0 \\ 0 & 0 & 0 & 0 & 1 & 0 \\ 0 & 0 & 0 & 0 & 0 & 1 \end{bmatrix} \begin{bmatrix} 0 \\ 0.3 \\ 0 \\ 0.1 \\ 0.4 \\ 0.2 \end{bmatrix}$$

Figure S1: **Deconvolution of a mixture where two aggregate signal frequencies are equal.** In this example, five aberrations (A_2, A_3, A_4, A_5 and A_6) were measured from an aggregate sample and their frequencies were $y_2 = 0.8$, $y_3 = 0.5$, $y_4 = 0.5$, $y_5 = 0.4$ and $y_6 = 0.2$, respectively. The dummy measurement $y_1 = 1$ was also added to generate the aggregate signal frequency vector $\mathbf{y} = [1, 0.8, 0.5, 0.5, 0.4, 0.2]$. In this example, there are two optimal TrAp solutions (left and right), each shown both as an evolutionary tree (top) and in matrix form according to Equation (**1**) (bottom). Both solutions have 4 common populated subclones, namely C_2 with aberration $\{A_2\}$, C_5 with aberrations $\{A_2, A_3, A_4, A_5\}$, C_6 with aberration $\{A_6\}$ and a subclone with aberrations $\{A_2, A_3, A_4\}$. In both cases, the ancestors of the clone with aberrations $\{A_2, A_3, A_4\}$ (C_3 of the left tree and C_4 of the right tree) are not populated. We remark that these two solutions are practically indistinguishable and that the TrAp algorithm outputs only the solution where the subclonal indices along each branch are arranged in an increasing order (as shown in the left tree solution).

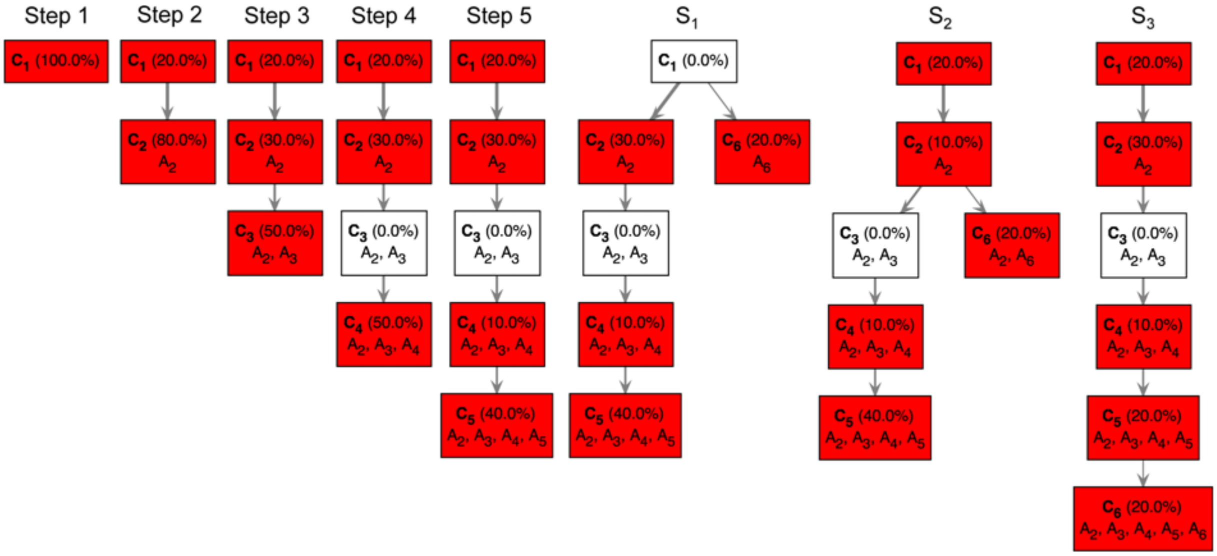

Figure S2: **Brute-force search approach to deconvolve a mixture of four subclones.** In this example, five aberrations (A_2, A_3, A_4, A_5 and A_6) were measured from an aggregate sample and their frequencies were $y_2 = 0.8$, $y_3 = 0.5$, $y_4 = 0.5$, $y_5 = 0.4$ and $y_6 = 0.2$, respectively. The dummy measurement $y_1 = 1$ was also added to generate the aggregate signal frequency vector $\mathbf{y} = [1, 0.8, 0.5, 0.5, 0.4, 0.2]$. In the first step, the wild type clone C_1 (representing the wildtype subpopulation) is positioned at the root of the tree. In the second step, a tree reconstruction begins and C_2 is added to the only possible ancestor clone C_1. In the third step, C_3 must be added as direct descendant of C_2. C_3 cannot be added to the tree on a different branch as a direct descendant of C_1 because based on Equation (**2**) this would imply a negative frequency $x_1 = y_1 - y_2 - y_3 = -0.3$. Likewise, the subclones C_4 and C_5 can only be added as direct descendants of C_3 and C_4, respectively. Finally, C_6 can be added as a direct descendant of subclones C_1, C_2 or C_5 generating solutions S_1, S_2 or S_3, respectively. However, S_1 is the only TrAp-solution as its number of populated subclones is minimal and corresponds the solution shown in the left side of Figure S1. Solution S_3 is the cascade-like solution described in Equation (**S1**).

both aberrations could be nonzero), we only output the solution for which $C_{\min\{i,j\}}$ is an ancestor of $C_{\max\{i,j\}}$ (left solution in Figure S1). This choice ensures that for every pair of aberrations $i < j$, C_j cannot be an ancestor of C_i. A step-by-step example of the TrAp solution obtained using the brute-force algorithm is given in Figure S2.

C Expressing the subclonal deconvolution problem as a function of the direct descendants

In Equation (**S2**), we expressed the aggregate frequency y_i as the sum of the frequencies of all subclones descending from C_i. We now express the aggregate frequency y_i as a function of the direct descendants of C_i. We define the **parent indicator variable** ϕ_{ij}, which is 1 if C_i is the parent of C_j (i.e. if subclone C_j is the result of a single aberration event in subclone C_i) and 0 otherwise. Finally, using the parent indicator variables we express y_i in terms of the aggregate frequencies of the direct descendants of C_i

$$\begin{aligned} y_i &= x_i + \sum_{j=1}^{N} \alpha_{ij} x_j = x_i + \sum_{j=1}^{N} \phi_{ij} \left(x_j + \sum_{k=1}^{N} \alpha_{jk} x_k \right) \\ &= x_i + \sum_{j=1}^{N} \phi_{ij} y_j, \end{aligned} \tag{S3}$$

Equation (**S3**) can be rearranged to express the vector $\mathbf{x}$ in terms of $\mathbf{y}$ and the parent indicator matrix $\mathbf{\Phi}$ (Equation (**2**) in the main text):

$$\mathbf{x} = \mathbf{y} - \mathbf{\Phi y},$$

where $\mathbf{\Phi}$ is the $N \times N$ matrix whose elements are given by the parent indicator variables $\phi_{i,j}$. Because of the evolutionary constraint, the matrix $\mathbf{\Phi}$ has only $N-1$ nonzero elements, reflecting the fact that each subclone except the wildtype has exactly one parent subclone, i.e. $\sum_{i=1}^{N} \phi_{i1} = 0$ and $\forall j > 1$, $\sum_{i=1}^{N} \phi_{ij} = 1$. Furthermore, an important corollary of Equation (**S3**) is that the subclone C_i is not populated if and only if (Equation (**3**) in the main text)

$$y_i - \sum_{j=1}^{N} \phi_{ij} y_j = 0.$$

In other words, the clone C_i is not populated when the aggregate frequency y_i of aberration i is equal to the sum of the aggregate frequencies of all the direct descendants of the subclone C_i. Therefore, the number of non-populated subclones of the N-tree encoded by $\mathbf{\Phi}$ is given by the number of aberrations i that satisfy Equation (**3**).

Finally, we summarize the relationships between $\mathbf{C}$ and the indicator variables α and ϕ. Using Equation (**S2**), we can express the subclonal deconvolution problem as $\mathbf{y} = \mathbf{Cx} = (\mathbf{I} + \mathbf{A})\mathbf{x}$, where $\mathbf{I}$ is the $N \times N$ identity matrix and $\mathbf{A}$ is the $N \times N$ matrix of whose elements are given by the ancestor indicator variable $\alpha_{i,j}$. Furthermore, Equation (**2**) allows us to write the subclonal deconvolution problem as $\mathbf{x} = (\mathbf{I} - \mathbf{\Phi})\mathbf{y}$. We can therefore express the relationships between the matrices $\mathbf{C}$, $\mathbf{A}$ and $\mathbf{\Phi}$ as:

$$\mathbf{C} = (\mathbf{I} + \mathbf{A}) = (\mathbf{I} - \mathbf{\Phi})^{-1}. \tag{S4}$$

We note that the parent indicator matrix $\mathbf{\Phi}$ and $\mathbf{C}$ are upper triangular and that both $\mathbf{C}$ and $\mathbf{I} - \mathbf{\Phi}$ are of rank N and invertible, which guarantees that Equation (**S4**) can always be used to switch between the representation with the parent indicator variable $\mathbf{\Phi}$ (Equation (**2**)) and the representation with the subclone matrix $\mathbf{C}$ (Equation (**1**)). We also note that, given a matrix $\mathbf{\Phi}$ (or $\mathbf{C} = (\mathbf{I} - \mathbf{\Phi})^{-1}$), the vector $\mathbf{x}$ is uniquely determined. In particular, if the $\mathbf{C}$ matrix is known, the vector $\mathbf{x}$ can be efficiently found by solving the linear system $\mathbf{Cx} = \mathbf{y}$ using back-substitution (i.e. by solving Equation (**S2**) first for x_N, then using x_N to solve for x_{N-1} and repeating through x_1).

D Generalization of TrAp to non-binary aberrations

In the previous sections we represented the genome of each subclone by a vector of binary values whose entries represent if a genomic position is in a normal state (0) or in an aberrant state (1). In general the number of states in a given genomic position could be larger than two and hence subclones cannot be represented by vectors of binary values without loss of information. For example, a nucleotide found in the reference genome or in the germline at a specific position may undergo multiple distinct point mutation events into more than one specific nucleotide. In this subsection, we describe an extension of the TrAp algorithm to deal with such cases.

We consider the **generalized subclonal deconvolution problem** in which the genome consists of N positions each of which can have S different states. We also assume that the genome of the wildtype subclone is known. The only information that we utilize as input is the **aggregate frequency matrix** $\mathbf{Z}$ whose elements $z_{k,s}$ correspond to the observed frequency of the aberrant state s at position k. We note that, by construction, $0 \leq z_{k,s} \leq 1$ and that $\sum_{s=1}^{S} z_{ks} = 1$. To utilize our framework for solving the subclonal deconvolution problem, we convert the information encoded in $\mathbf{Z}$ as a vector $\mathbf{y}$ whose elements represent frequencies of binary events. We perform this transformation in several steps. First, we vectorize the matrix $\mathbf{Z}$ by concatenating its rows to construct the vector $\mathbf{z}$, which has KS elements. Then, we remove from the vector $\mathbf{z}$ the entries for which $z_{ks} = 0$ as they are not informative. As a result, for each position k there are S_k entries, where S_k ranges from 1 (only the unmutated state is observed) to S (all aberrant states are observed). For illustration of these first steps, we consider a toy example of a genome of length three whose wildtype sequence is "CAT" and we analyze an aggregate sample made of three subclones with sequences "TCT", "TAC" and "CGT" mixed with frequencies 0.1, 0.3 and 0.6, respectively (Figure S3). In this example the $\mathbf{z}$ vector consists of the elements $z_{1C} = 0.6$, $z_{1T} = 0.4$, $z_{2A} = 0.3$, $z_{2C} = 0.1$, $z_{2G} = 0.6$, $z_{3C} = 0.3$ and $z_{3T} = 0.7$ (Figure S3).

Next, we wish to design a binarization matrix $\mathbf{B}$ to encode the information contained in $\mathbf{z}$ as a vector $\mathbf{y} = \mathbf{Bz}$ whose elements represent the frequency of binary aberrations and can thus be used as input to the subclonal deconvolution problem for the whole genome (Equation (**1**)). For every position k, we assume that each state s ($1 \leq s \leq S_k$) is reached by a sequence of aberration events. We denote by A_{ks} an aberration event to state s at position k. In the example above, there are two states at position 1. The unmutated state C is reached by a dummy aberration A_{1C} (in analogy to the dummy aberration of the wildtype clone in the subclonal deconvolution problem) and the aberrant state T is reached by the sequence of the dummy aberration A_{1C} followed by the aberration A_{1T}. Since the dummy aberration A_{1C} is present when we observe states C or T at position 1, the frequency of the unmutated aberration is $y_{1C} = z_{1C} + z_{1T} = 1$. However, the aberration A_{1T} is present only when we observe the state T, therefore the frequency of the aberration A_{1T} is $y_{1T} = z_{1T} = 0.4$ (Figure S3). Since measurements at different genomic positions do not affect one another, we construct $\mathbf{B}$ as a block-diagonal matrix:

$$\begin{bmatrix} \mathbf{y_1} \\ \hline \mathbf{y_2} \\ \hline \vdots \\ \hline \mathbf{y_K} \end{bmatrix} = \begin{bmatrix} \mathbf{B_1} & 0 & \cdots & 0 \\ 0 & \mathbf{B_2} & \cdots & 0 \\ \vdots & \vdots & \ddots & \vdots \\ 0 & 0 & \cdots & \mathbf{B_K} \end{bmatrix} \begin{bmatrix} \mathbf{z_1} \\ \hline \mathbf{z_2} \\ \hline \vdots \\ \hline \mathbf{z_K} \end{bmatrix}, \quad (\mathbf{S5})$$

where $\mathbf{y_k} = [y_{k1}, \ldots, y_{kS_k}]$ and $\mathbf{z_k} = [z_{k1}, \ldots, z_{kS_k}]$. Combining Equation (**1**) and Equation (**S5**) gives

$$\mathbf{Bz} = \mathbf{Cx}. \quad (\mathbf{S6})$$

It is important to note that for any pair of different states s_1 and s_2 at position k where $1 \leq s_1 \leq S_k$ and $1 \leq s_2 \leq S_k$, the value of b_{ks_1,ks_2} defines the ancestral relationship between the aberration events A_{ks_1} and A_{ks_2}. Using the ancestor indicator variable α we can express this relationship as $\alpha_{ks_1,ks_2} = b_{ks_1,ks_2}$. To preserve these ancestral relationships in both sides of Equation (**S6**) (we recall that $\mathbf{C} = \mathbf{I} + \mathbf{A}$), the

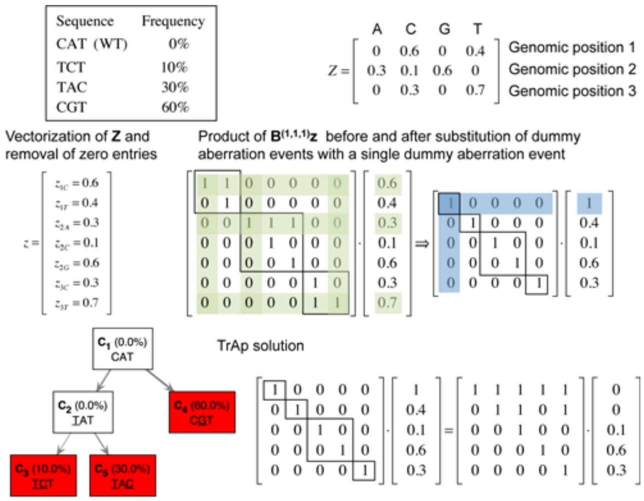

Figure S3: **Application of the TrAp algorithm to deconvolve a mixture of three sequences in presence of poly-allelic mutations.** We analyzed an aggregate sample composed of three subclones with sequences "TCT", "TAC" and "CGT" mixed with frequencies 0.1, 0.3 and 0.6, respectively. In this particular example, the wildtype sequence "CAT" is absent in the mixture. Nonzero elements of the aggregate frequency matrix $\mathbf{Z}$ (top right) are then concatenated in the $\mathbf{z}$ vector, which consists of the elements $z_{1C} = 0.6$, $z_{1T} = 0.4$, $z_{2A} = 0.3$, $z_{2C} = 0.1$, $z_{2G} = 0.6$, $z_{3C} = 0.3$ and $z_{3T} = 0.7$ (middle left). In the center of the middle panel we show the binarization matrix $\mathbf{B}^{(1,1,1)}$ and the matrix-vector product $\mathbf{B}^{(1,1,1)}\mathbf{z}$ associated with it, which are consistent with Equation (**S5**) and lead to the optimal TrAp solution. Next, rows and columns corresponding to unmutated states (i.e. $1C$, $2A$ and $3T$, shown in green) are substituted with a dummy aberrant state corresponding to the wildtype (shown in blue). In the bottom row, the TrAp solution is shown both as an evolutionary tree (left) and in matrix form according to Equation (**S6**) (right).

element c_{ks_1,ks_2} of $\mathbf{C}$ must be equal to the element b_{ks_1,ks_2} of $\mathbf{B}$ for every pair of states s_1 and s_2 at position k, where $1 \leq k \leq K$, $1 \leq s_1 \leq S_k$ and $1 \leq s_1 \leq S_k$.

In order to find the solutions to Equation (**S5**), we solve independently each subproblem $\mathbf{y_k} = \mathbf{B_k z_k}$ and we require that the $\mathbf{B_k}$ matrix encodes a tree which satisfies the evolutionary and parsimony constraints and whose root is the dummy unmutated aberration at position k. The number of solutions for each subproblem is equal to the number of trees with S_k labeled vertices and, as discussed earlier, this number is equal to $S_k^{S_k-2}$. We then denote by $\mathbf{B_k^{(t_k)}}$ the t_k-th solution ($1 \leq t_k \leq S_k^{S_k-2}$) and by $\mathbf{y_k^{(t_k)}}$ the aggregate frequency vector associated to it. The solutions to the problem for $S_k \leq 3$ are:

$S_k = 1$. There is only one solution. Since the input consists only of the unmutated state, it follows that $\mathbf{z_k} = [1]$. The solution is $\mathbf{B_k^{(1)}} = [1]$ and the corresponding aggregate frequency vector is $\mathbf{y_k^{(1)}} = [1]$.

$S_k = 2$. There is only one solution. Assuming the input is $\mathbf{z_k} = [z_{k1}, z_{k2}]$ and the unmutated state is $k1$, the solution is $\mathbf{B_k^{(1)}} = \begin{bmatrix} 1 & 1 \\ 0 & 1 \end{bmatrix}$ and the corresponding aggregate frequency vector is $\mathbf{y_k^{(1)}} = [1, z_{k2}]$.

$S_k = 3$. There are 3 solutions. Assuming the input is $\mathbf{z_k} = [z_{k1}, z_{k2}, z_{k3}]$ and unmutated state is $k1$, the solutions are $\mathbf{B_k^{(1)}} = \begin{bmatrix} 1 & 1 & 1 \\ 0 & 1 & 0 \\ 0 & 0 & 1 \end{bmatrix}$, $\mathbf{B_k^{(2)}} = \begin{bmatrix} 1 & 1 & 1 \\ 0 & 1 & 1 \\ 0 & 0 & 1 \end{bmatrix}$ and $\mathbf{B_k^{(3)}} = \begin{bmatrix} 1 & 1 & 1 \\ 0 & 1 & 0 \\ 0 & 1 & 1 \end{bmatrix}$ and their

corresponding $\mathbf{y_k}$ vectors are given by $\mathbf{y_k^{(1)}} = [1, z_{k2}, z_{k3}]$, $\mathbf{y_k^{(2)}} = [1, z_{k2} + z_{k3}, z_{k3}]$ and $\mathbf{y_k^{(3)}} = [1, z_{k2}, z_{k2} + z_{k3}]$. The values $b_{k2,k3}$ and $b_{k3,k2}$ define the ancestral relationship between aberrations A_{k2} and A_{k3}. In the first solution A_{k2} and A_{k3} must be on separate branches, in the second solution A_{k2} must be an ancestor of A_{k3}, and in the third solution A_{k3} must be an ancestor of A_{k2}.

The number of solutions grows dramatically with S_k (16 solutions for $S_k = 4$, 125 solutions for $S_k = 5$, 1296 solutions for $S_k = 6$). Therefore, herein we explicitly show the solutions for $S_k < 4$ and implement the method to adress practical scenarios, such as nucleotide point mutations ($S_k \leq 4$).

The set of all possible solutions to Equation (**S5**) is given by the Cartesian product of the solution sets of each subproblem. This implies that the number of solutions of Equation (**S5**) is $\prod_{k=1}^{K} S_k^{S_k - 2}$. We denote by $\mathbf{B^{(t_1,\dots,t_K)}}$ the block matrix solution associated with the blocks $\mathbf{B_1^{t_1}}, \dots, \mathbf{B_K^{t_K}}$ representing the solutions of each subproblem. Similarly, we denote by $\mathbf{y^{(t_1,\dots,t_K)}}$ the aggregate signal vector given by $\mathbf{y^{(t_1,\dots,t_K)}} = \mathbf{B^{(t_1,\dots,t_K)}}\mathbf{z}$. It is possible to reduce the number of rows and columns in Equation (**S6**) by substituting all the K rows corresponding to the unmutated states (shown in green in Figure S3) with a single dummy wildtype aberration (shown in blue in Figure S3). In our toy example, one aberrant state is observed at positions 1 and 3 ($S_1 = S_3 = 2$), while two aberrant states (C and G) are observed at position 2 ($S_2 = 3$). Therefore, there are three solutions to Equation (**S5**): $\mathbf{B^{(1,1,1)}}$, $\mathbf{B^{(1,2,1)}}$ and $\mathbf{B^{(1,3,1)}}$. Figure S4 illustrates the best solution for each of these binarization matrices in a graphical and matrix form. The solution associated with $\mathbf{B^{(1,1,1)}}$ is the only TrAp-solution of the generalized subclonal deconvolution problem since it has the minimum number of populated subclones (sparsity constraint).

In summary the generalized subclonal deconvolution problem can be solved as follows:

1. Vectorize the aggregate frequency matrix $\mathbf{Z}$ and identify all binarization matrices $\mathbf{B^{(t_1,\dots,t_K)}}$ (Equation (**S5**)) compatible with the vector $\mathbf{z}$.

2. For each binarization matrix $\mathbf{B^{(t_1,\dots,t_K)}}$, identify all first generation trees from the aggregate signal vector $\mathbf{y^{(t_1,\dots,t_K)}} = \mathbf{B^{(t_1,\dots,t_K)}}\mathbf{z}$ and combine the first generation trees to generate all partial trees compatible with $\mathbf{B^{(t_1,\dots,t_K)}}$.

3. Discard partial trees that do not have the minimum number of populated subclones.

4. Generate all evolutionary trees consistent with the partial trees comprising a maximum number of first generation trees. This step is performed as described above, but with the additional constraint that $c_{ks1,ks2} = b_{ks1,ks2}$ for any pair of states s_1 and s_2 at position k, where $1 \leq k \leq K$, $1 \leq s_1 \leq S_k$ and $1 \leq s_2 \leq S_k$.

5. Optimize the shallowness constraint by sorting the generated solutions by the depth of the generated tree.

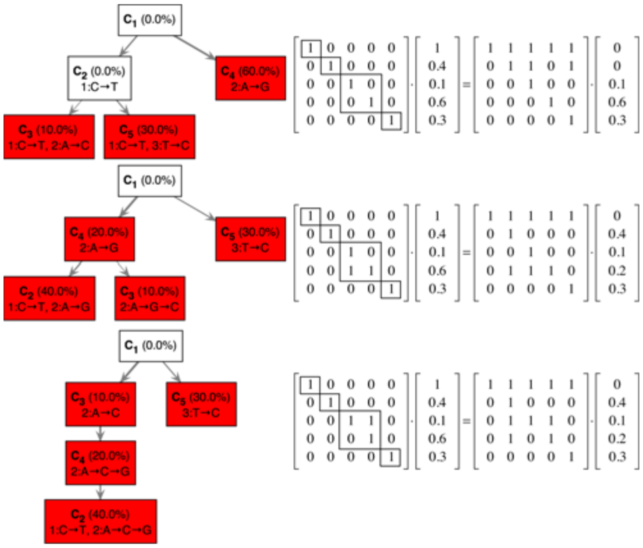

Figure S4: **Three solutions of the generalized subclonal deconvolution problem for a mixture of three sequences in presence of poly-allelic mutations.** We analyzed an aggregate sample composed of three subclones with sequences "TCT", "TAC" and "CGT" mixed with frequencies 0.1, 0.3 and 0.6, respectively. In this example, the wildtype sequence "CAT" is absent in the mixture. Nonzero elements of the aggregate frequency matrix $\mathbf{Z}$ are concatenated in the $\mathbf{z}$ vector, which consists of the elements $z_{1C} = 0.6$, $z_{1T} = 0.4$, $z_{2A} = 0.3$, $z_{2C} = 0.1$, $z_{2G} = 0.6$, $z_{3C} = 0.3$ and $z_{3T} = 0.7$. There are three binarization matrices ($\mathbf{B}^{(\mathbf{1},\mathbf{1},\mathbf{1})}$, $\mathbf{B}^{(\mathbf{1},\mathbf{2},\mathbf{1})}$ and $\mathbf{B}^{(\mathbf{1},\mathbf{3},\mathbf{1})}$) to Equation (**S5**) and one solution for each binarization matrix is shown. Mutations are shown using the notation "position : reference → mutated", e.g. the notation $2 : A \to G$ indicates that nucleotide at position 2 was mutated from Adenine to Guanine. The notation $2 : A \to G \to C$ indicates that nucleotide at position 2 was first mutated from Adenine to Guanine and then from Guanine to Cytosine. Top: solution based on the binarization matrix $\mathbf{B}^{(\mathbf{1},\mathbf{1},\mathbf{1})}$, in which the subclones C_3 and C_4 associated with the aberration events A_{2C} and A_{2G} are on separate branches; Middle: solution based on the binarization matrix $\mathbf{B}^{(\mathbf{1},\mathbf{2},\mathbf{1})}$, in which the aberration event A_{2G} (subclone C_4) happens before of A_{2C} (subclone C_3; bottom: solution based on the binarization matrix $\mathbf{B}^{(\mathbf{1},\mathbf{3},\mathbf{1})}$, in which the aberration event A_{2C} (subclone C_3) happens before A_{2G} (clone C_4). The solutions are shown both as evolutionary trees (left) and in matrix form according to Equation (**S6**).

Supplementary Figures

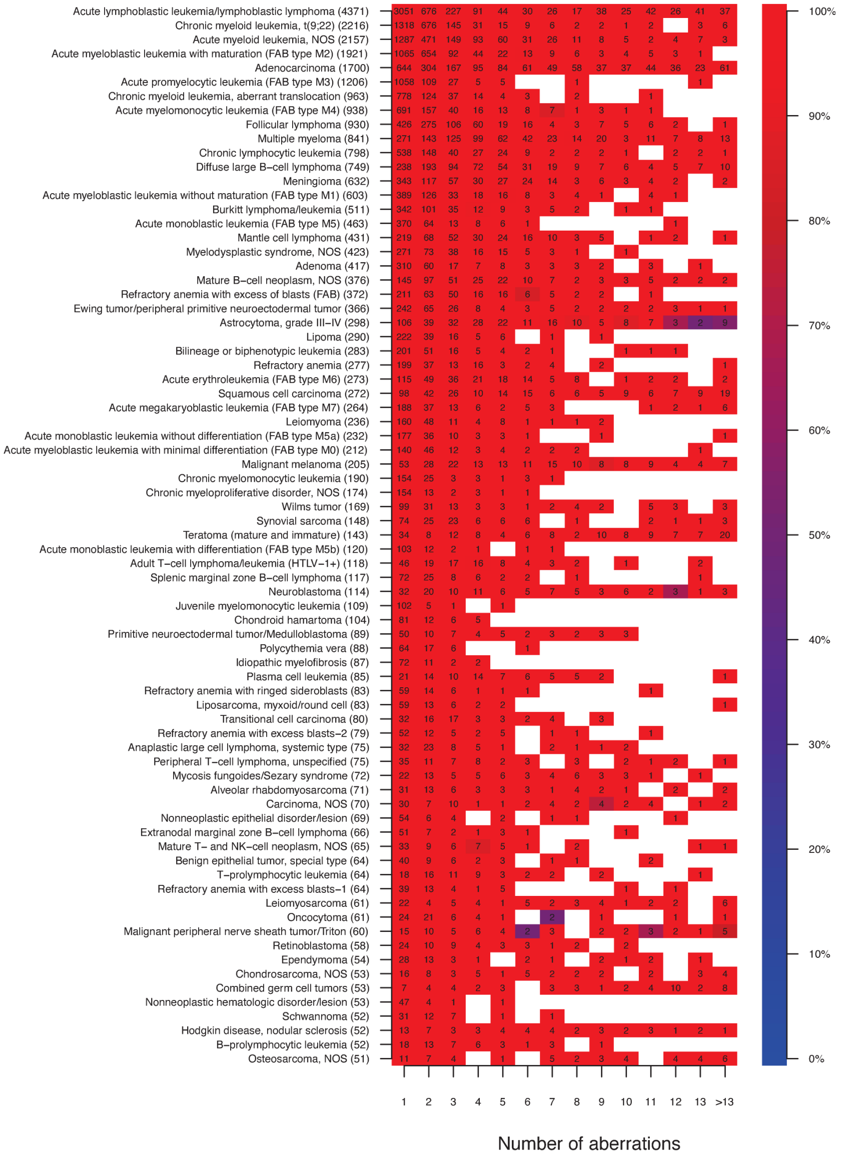

Figure S5: **Applicability of the TrAp algorithm for different number of aberrations events and different kind of tumors.** Each entry in the heat map shows the number of biopsies, which contained a given number of aberrations. Each cell is colored by the fraction of biopsies for which TrAp could reconstruct the correct composition of the subclones, from red (all biopsies could be reconstructed) to blue (no biopsies could be reconstructed). The constraints required by the TrAp algorithm are satisfied in most cancer types, with the exception of astrocytoma of grades III and IV.

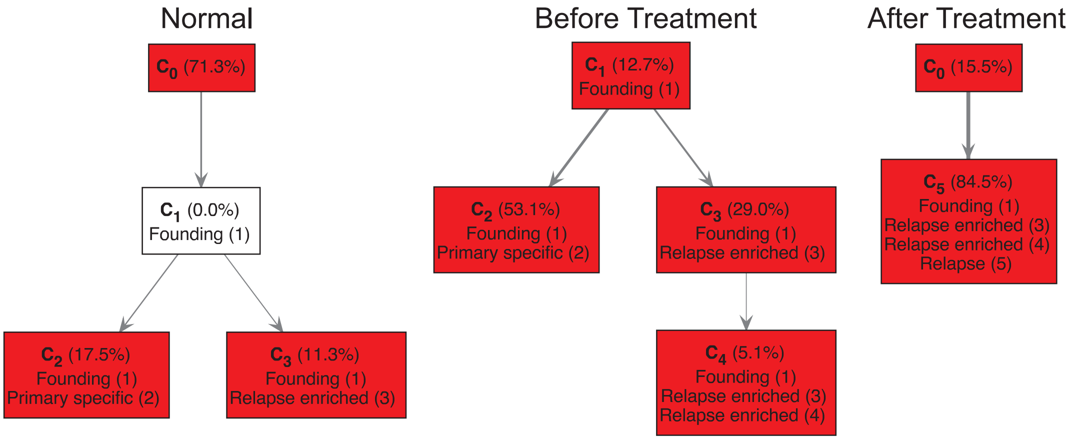

Figure S6: **Inference of the clonal evolution of the AML patient UPN933124.** The data shows that after treatment, only the subclone C_4 survived and acquired new mutations to form C_5. We note that the normal sample is contaminated with tumor tissue ($\sim$29%), the sample before treatment is not significantly contamined with normal tissue and that the sample after treatment is contaminated with normal tissue ($\sim$15%). The data and the clusters of mutations were obtained from Ding *et al.* [18] and analyzed using the TrAp algorithm.

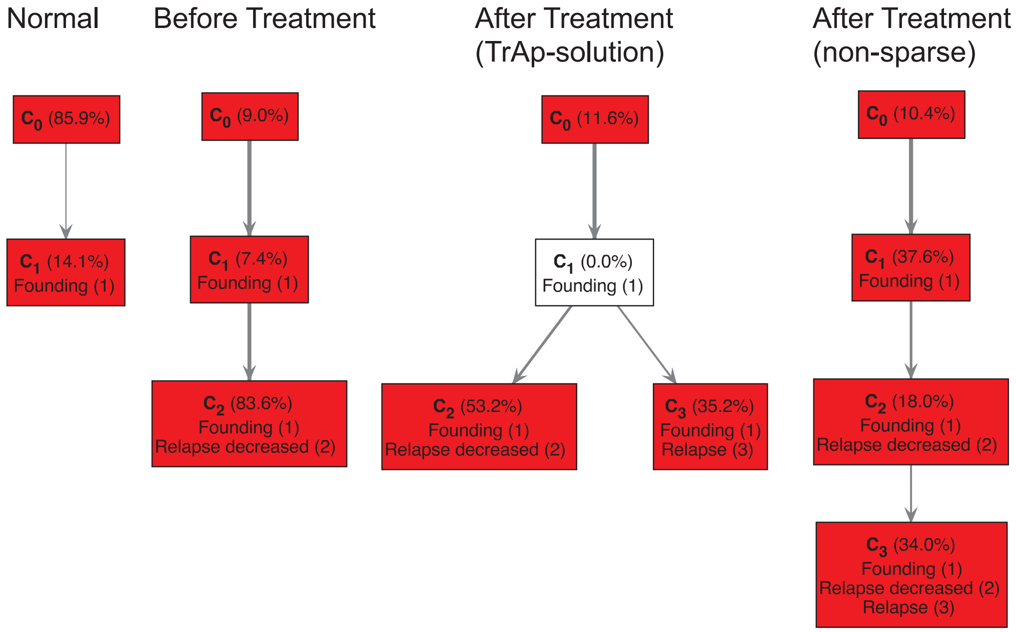

Figure S7: **Inference of the clonal evolution of AML patient UPN758168.** The data shows that after treatment, the founding subclone C_1 aquired new mutations to form a new relapse sublclone C_3 and that the relative size of subclone C_2 was significantly reduced. The TrAp software also returned a suboptimal non-sparse solution for the sample after treatment (on the right), where the relapse subclone C_3 originated from subclone C_2 instead of C_1. The data and the clusters of mutations were obtained from Ding *et al.* [18] and analyzed using the TrAp algorithm.

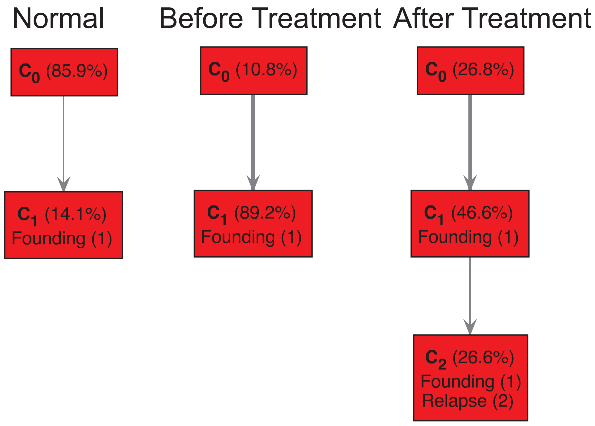

Figure S8: **Inference of the clonal evolution of AML patient UPN400220.** The data shows that, after treatment, the founding subclone C_1 aquired new mutations to form a new relapse sublclone C_2.The data and the clusters of mutations were obtained from Ding *et al.* [18] and analyzed using the TrAp algorithm.

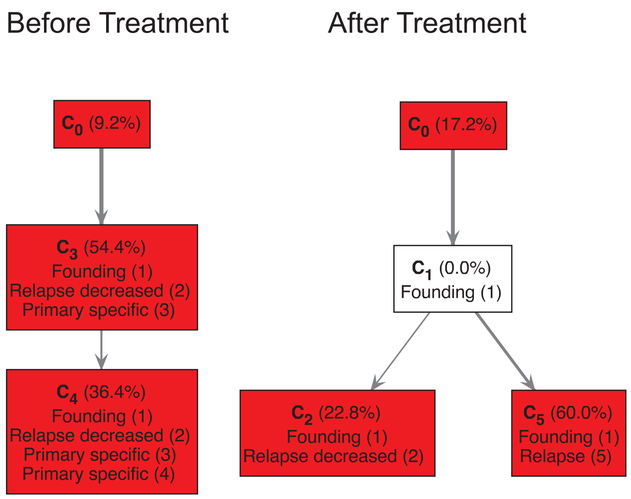

Figure S9: **Inference of the clonal evolution of AML patient UPN426980.** The data shows that after treatment, the founding subclone C_1 aquired new mutations to form a new relapse sublclone C_5 and that the primary specific subclones C_3 and C_4 were eradicated. The sample from the normal tissue is not shown as this sample is not significantly contaminated by the tumor. The data and the clusters of mutations were obtained from Ding *et al.* [18] and analyzed using the TrAp algorithm.

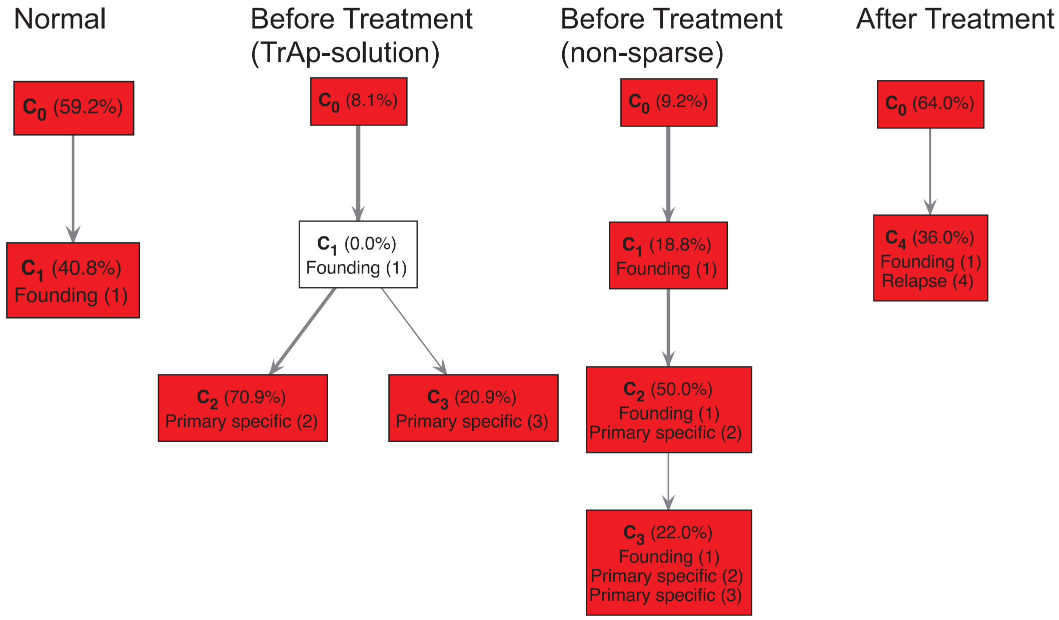

Figure S10: **Inference of the clonal evolution of AML patient UPN452198.** The data shows that after treatment, the founding subclone C_1 aquired new mutations to form a new relapse sublclone C_4 and that the primary specific subclones C_2 and C_3 were eradicated. We also show a suboptimal non-sparse solution for the sample before treatment, where the primary specific subclone C_3 originated from subclone C_2 instead of C_1. The data and the clusters of mutations were obtained from Ding *et al.* [18] and analyzed using the TrAp algorithm.

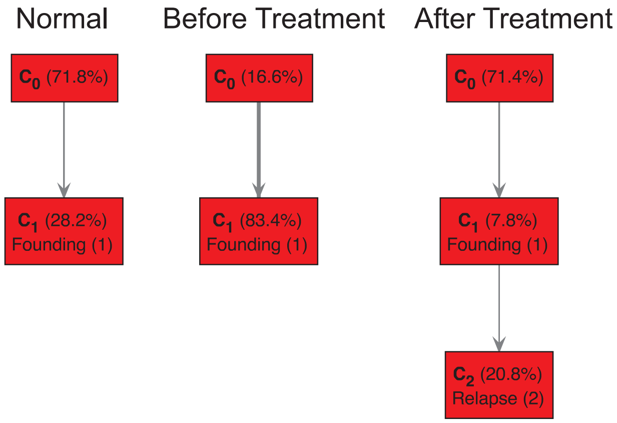

Figure S11: **Inference of the clonal evolution of AML patient UPN573988.** The data shows that, after treatment, the founding subclone C_1 aquired new mutations to form a new relapse sublclone C_2. The data and the clusters of mutations were obtained from Ding *et al.* [18] and analyzed using the TrAp algorithm.

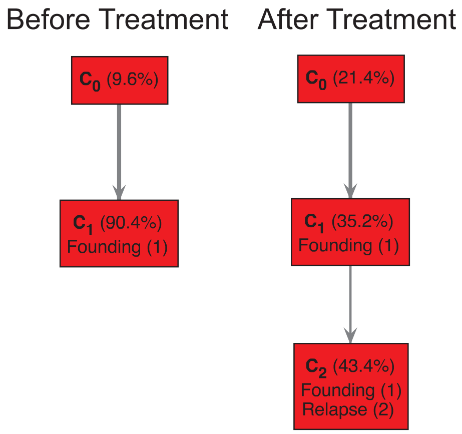

Figure S12: **Inference of the clonal evolution of AML patient UPN804168.** The data shows that, after treatment, the founding subclone C_1 aquired new mutations to form a new relapse sublclone C_2. The sample from the normal tissue is not shown as this sample is not significantly contaminated by the tumor. The data and the clusters of mutations were obtained from Ding *et al.* [18] and analyzed using the TrAp algorithm.

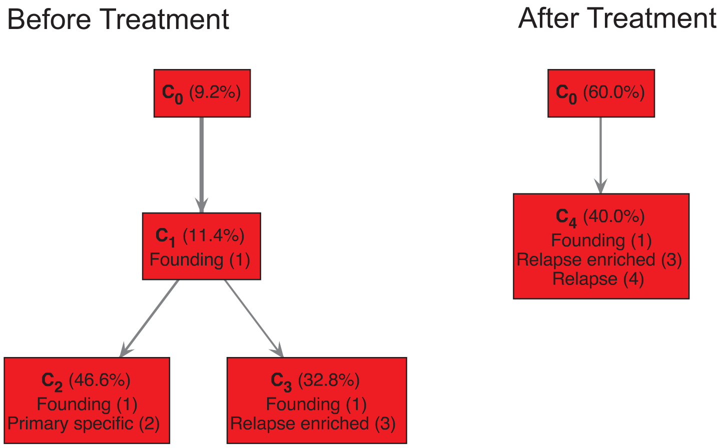

Figure S13: **Inference of the clonal evolution of AML patient UPN869586.** The data shows that, after treatment, the subclone C_3 aquired new mutations to form a new relapse sublclone C_4, while the subclone C_2 was eradicated. The sample from the normal tissue is not shown as this sample is not significantly contaminated by the tumor. The sample from the normal tissue is not shown as this sample is not significantly contaminated by the tumor. The data and the clusters of mutations were obtained from Ding *et al.* [18] and analyzed using the TrAp algorithm.

References

[18] Ding, L., Ley, T.J., Larson, D.E., Miller, C.A., Koboldt, D.C., Welch, J.S., Ritchey, J.K., Young, M.A., Lamprecht, T., McLellan, M.D. *et al.* (2012) Clonal evolution in relapsed acute myeloid leukaemia revealed by whole-genome sequencing. *Nature*, **481**, 506-510.
